# Quality of maternal and newborn health care in Ethiopia: a cross-sectional study

**DOI:** 10.1186/s12913-021-06680-1

**Published:** 2021-07-10

**Authors:** Abera Biadgo, Aynalem Legesse, Abiy Seifu Estifanos, Kavita Singh, Zewdie Mulissa, Abiyou Kiflie, Hema Magge, Befikadu Bitewulign, Mehiret Abate, Haregeweyni Alemu

**Affiliations:** 1 Institute for Healthcare Improvement, Ethiopia Project Office, Addis Ababa, Ethiopia; 2 Ministry of health of Ethiopia, Addis Ababa, Ethiopia; 3 Department of Reproductive, Family and Population Health, School of Public Health, College of Health Sciences, Addis Ababa University, Addis Ababa, Ethiopia; 4 Department of Maternal and Child Health, Gillings School of Global Public Health, University of North Carolina at Chapel Hill, 135 Dauer Dr, Chapel Hill, NC 27599 USA; 5 Brigham and Women’s Hospital Division of Global Health Equity, Boston, USA

**Keywords:** MNH quality of care, Health facilities, Input, Process, Output, Ethiopia

## Abstract

**Background:**

Despite reports of universal access to and modest utilization of maternal and newborn health services in Ethiopia, mothers and newborns continue to die from preventable causes. Studies indicate this could be due to poor quality of care provided in health systems. Evidences show that high quality health care prevents more than half of all maternal deaths. In Ethiopia, there is limited knowledge surrounding the status of the quality of maternal and newborn health care in health facilities. This study aims to assess the quality of maternal and neonatal health care provision at the health facility level in four regions in Ethiopia.

**Methodology:**

This study employed a facility-based cross-sectional study design. It included 32 health facilities which were part of the facilities for prototyping maternal and neonatal health quality improvement interventions. Data was collected using a structured questionnaire, key informant interviews and record reviews. Data was entered in Microsoft Excel and exported to STATA for analysis. Descriptive analysis results are presented in texts, tables and graphs. Quality of maternal and neonatal health care was measured by input, process and outputs components. The components were developed by computing scores using standards used to measure the three components of the quality of maternal and neonatal health care.

**Result:**

The study was done in a total of 32 health facilities: 5 hospitals and 27 health centers in four regions. The study revealed that the average value of the quality of the maternal and neonatal health care input component among health facilities was 62%, while the quality of the process component was 43%. The quality of the maternal and neonatal health output component was 48%. According to the standard cut-off point for MNH quality of care, only 5 (15.6%), 3 (9.3%) and 3 (10.7%) of health facilities met the expected input, process and output maternal and neonatal health care quality standards, respectively.

**Conclusion:**

This study revealed that the majority of health facilities did not meet the national MNH quality of care standards. Focus should be directed towards improving the input, process and output standards of the maternal and neonatal health care quality, with the strongest focus on process improvement.

**Supplementary Information:**

The online version contains supplementary material available at 10.1186/s12913-021-06680-1.

## Introduction

Worldwide, over 70%, of maternal deaths are caused by pregnancy related complications including hemorrhages, pregnancy related hypertension, sepsis and abortion. Similarly, about 85% of newborn deaths are due to birth prematurity, asphyxia, and neonatal infections [1].

In Ethiopia, notable efforts have been made to improve access to basic and emergency obstetric and newborn care such as expansion of health facilities and availing BEmONC and CEmONC services. However, there is inadequate utilization of pregnancy and childbirth care services. Population-level coverage of maternal health services range from 74% for first antenatal care (ANC) visit to 43% for four or more ANC visits, 48% for skilled birth attendants (SBA) and 34% for postnatal care (PNC) within 2 days after birth [2]. That leads to the least progress on reducing neonatal mortality which stands at 29 deaths per 1000 live births in 2016 and 30 in 2019 [2, 3].

Increasing access to and utilization of services alone does not guarantee improved maternal and newborn health outcomes [4]. Along with expanding coverage of the services improving quality and evidence-based care during critical periods will have the greatest impact on the survival of the mother, the fetus and the newborn [4, 5]. The World Health Organization (WHO) defines quality of care as “the extent to which health care services provided to individuals and patient populations improve desired health outcomes [6]. In order to achieve this, health care must be safe, effective, timely, efficient, equitable and people-centered” [7]. Quality of care improvement requires availing skilled birth attendants for every pregnant woman and newborn, and evidence-based care provision including respectful care, creating supportive environment, use of effective clinical and non-clinical interventions, health care infrastructure capacity, health care providers skills and positive attitude [4, 5].

In 2016, Ethiopia adapted WHO standards for improving quality of maternal and newborn health care in health facilities and developed health sector transformation in quality (HSTQ) [8] which helps to facilitate implementation of national quality strategy (NQS). The NQS was developed in 2015 and prioritizes maternal and newborn health as per the health sector transformation plan (HSTP) [9, 10] of the country. The HSTP is a five-year national strategic plan and has four transformation agenda/priorities (1. Transformation in Quality & Equity, 2. Woreda Transformation, 3. Information Revolution, and 4. Compassionate, Respectful and Caring Health Workers) [10]. One of the transformation agenda is ensuring quality and equity in the health care system and to achieve this transformation agenda, the NQS was developed. The aim of the NQS is to consistently improve the outcomes of clinical care, patient safety, and patient-centeredness, while increasing access and equity for all segments of the Ethiopian population; on top of that the MNH is the top priority in the strategy. Three core elements of quality were spelled out in the NQS, namely quality planning, quality improvement and quality control [9]. Following the NQS development a quality standard was developed to measure the quality of health care. That quality measure standard document is the HSTQ which is in line with WHO MNH quality standard [4, 8]. Everyone in the health system has their own responsibility to implement the NQS and HSTQ. The health facilities are responsible to measure, identify, analyze and solve their own quality issues, which in turn leads to provision of higher quality care [8, 9].

Implementing quality of health care which laid out in the NQS in Ethiopia, Institute for Healthcare Improvement (IHI) and Ministry of Health (MoH) have worked to demonstrate how quality improvement (QI) methodologies can be applied in the Ethiopian health care system to accelerate progress of maternal and neonatal health (MNH). The aim of the project was to strengthen and develop a sustainable, self-sufficient Ethiopian health care quality culture and capability across multiple levels of the health system; and launch and test a scalable model of health system improvement in health facilities and communities. The MOH across the multiple level would lead the process and IHI supports the health system to achieve the overall aim. To introduce quality improvement (QI) methods, a district-wide improvement collaborative approach [11] was applied. Collaborative is a short-term (12 to 18 months) learning system that brings together a large number of teams from health facilities to seek improvement in a focused topic area.

The prototype sites were quality health care collaborative demonstration and learning sites for the broader aim to scale up to all districts. Those prototype sites were four collaboratives and they practiced similar collaborative approach. Hence a baseline assessment of quality MNH care was done prior to the start of the improvement program that aimed to identify critical gaps that hinder the performance and improve the facilities’ performance. This paper describes the baseline quality of care of MNH in the prototype district facilities in the four regions in Ethiopia.

## Methods

### Study areas

Ethiopia has very diversified culture and more than 86 indigenous languages. Administratively, it has ten regions and two city administrations. Among those 10 regions and two city administration, the four regions which the four prototype districts selected house 81% of the country’s population. Each region subdivided into districts and there are over 850 districts in nationwide. Health service delivery is provided through a three-tier system as primary, secondary, and tertiary level health care.

Four districts were chosen for the prototype phase of the QI project that began in 2016: Limu bilbilu, Tanqua Abergele, Duguna Fango and Fogera in the Oromia, Tigray, Southern Nations, Nationalities and Peoples’ (SNNP) and Amhara regions, respectively. A total of 121 health posts, 27 health centers and 5 hospitals that were providing MNH care for the population in the four districts were included in prototype and clustered into four QI collaborative sites, one for each district. The catchment populations of the collaborative sites were 213,032 in Limu Bilbilu, 115,841 in Tanqua Abergele, 122,316 in Duguna Fango, and 296,842 in Fogera districts.

The prototype collaborative sites were purposefully selected in consultation with Ministry of Health of Ethiopia and regional health bureaus (RHBs) based on pre-set criteria. The criteria included high maternal and perinatal deaths, high level of leadership commitment to improve the service, reliability of MNH service data, and no other partner organizations working on quality improvement project in the sites to minimize duplication of efforts. All health facilities under the selected districts were included in the collaborative including health posts, health centers and hospitals. All districts had maternal mortality, still births and neonatal deaths; they are agrarian; the staff were interested to improve the system and no organization working on quality issues.

### Study design

A facility based cross-sectional study was deployed to determine the quality of MNH care in the QI collaborative sites. Quality of MNH care was measured using input, process and outcome components. The components were developed using input, process and output MNH quality standards of the WHO and HSTQ for health facilities of Ethiopia MoH. Data were collected in 2016 using face to face interviews and data extraction.

### Data collection technique and tools

Data were organized to be collected as elements of the input, process, and outcome variable. Input quality standards was developed using 28 items related to the infrastructure, supplies and equipment standards. Process quality standards was developed using 13 items of the labour, delivery and postnatal care provision and complication management standards. Outcome quality standards was measured using four items related to the health seeking behavior standards (Table 1). The three quality standard elements were selected and withdrawn from the broader HSTQ standard measures for the purpose of the study. Those all items of inputs, process and output standards were selected based on the national HSTQ MNH focus and their contribution to maternal and neonatal mortality.

**Table 1:** Quality standard items list

***Input Quality items***	
***Infrastructure***	***Essential drugs***
*Functional incinerator on the time of visit.*	*Dexamethasone/betamethasone IV*
*Functional placenta pit on the time of visit.*	*Methyldopa*
*Electric power supplies any type.*	*Hydralazine*
*Water availability in the compound either in the form of pipe water or hand pump.*	*Nifedipine*
***Equipment/supply***	*Ampicillin (IV)*
*Newborn corner in the delivery room.*	*Gentamycin (IV)*
*Baby Weighing scales for Newborns with 50 precision.*	*Metronidazole (IV)*
*Complete delivery sets (Kidney dish, forceps, gauze swabs, Scissors, pads, cord tie/clips) at least three per facility.*	*Tetracycline (TTC) eye ointment*
*Towel for baby drying and wrapping*	*Vitamin K*
*Bulb suction*	*MgSO4*
*Ambu bag & face masks (neonatal- size 0)*	*Calcium gluconate*
*Ambu bag & face masks (neonatal- size 1)*	*Oxytocin*
*Oxygen concentrator/Oxygen cylinder*	*Normal saline/Ringers lactate*
*Radiant warmer*	
*Running water at the delivery room.*	
*Soap at the delivery room.*	
***Process Quality items***	
***Clinical care for MNH***	***Complication management***
*Proportion of deliveries which danger signs assessed on admission (BP measured)*	*Proportion of women with pre-eclampsia who are treated with IV/IM MgS04*
*Proportion of deliveries which partograph started when cervical dilatation at least 4 cm*	*Proportion of pregnant women with pPRoM who are not in labor and are given oral erythromycin*
*Proportion of mothers who received 10 IU IV/IM Oxytocin*	*Proportion of Postpartum Hemorrhage cases managed per protocol*
*Proportion of newborn who had assessment (does the baby need special care and monitoring) – APGAR score*	*Proportion of asphyxiated neonates who were resuscitated (with bag & mask) and survived*
*Proportion of deliveries (newborns) who received immediate skin to skin and initiate breastfeeding within the 1st hour (measured by mother/baby bonding)*	*Proportion of Sick Young infants treated for sepsis/VSD*
*Proportion of deliveries (newborns) who received Vit K1*	*Proportion of low birth weight or premature newborns for whom KMC was initiated after delivery*
*Proportion of deliveries (newborns) who received Tetracycline Eye Ointment*	
***Output Quality items***	
*Proportion of women that received antenatal care four or more times during the current pregnancy*	*Proportion of births attended by skilled health personnel (midwife, nurse, health officer or doctor)*
*Proportion of pregnant women attending antenatal care clinics tested for syphilis*	*Proportion of births that received post-natal care at least once during the early post-partum period (within 48 h after delivery)*

The study included 32 health facilities which were part of the facilities for prototyping maternal and neonatal health quality improvement interventions. All health facility heads, and maternity care related department heads were included for the interviews. Data from individual patient records were extracted through selecting individual patient’s records by applying a systematic random sampling method using medical record numbers (MRN) as the sample frame. Previous 6 months delivery records in the MNH registration were included in the sample frame and it is sampled ten medical records for every month in each facility. There were 9602 medical records in total from all health facilities in the six-month period.

The data was collected at the start of the implementation of IHI’s project from Sep 2016 to Nov 2016 at the health facilities. Structured interviews with health facility and department heads were conducted to assess availability of resources. Direct observation was also done to complement and verify interview results to cross check the available infrastructures, medical equipment’s, supplies, and available services. Before the data collection the data collectors discussed and agreed upon each question. The data collectors were IHI staff and zonal health departments (ZHD)/district health offices MNH and HMIS officers. The collected data were cross-checked for completeness immediately after completion.

The data collection tool had two parts: 1) An interview guide which was used to collect the data from the health facility heads and maternity care related department heads. During the interview, there was cross-checking of records through direct looking on records to confirm the reliability of the data they provided. 2) A data abstraction form which was used to collect the data from the MNH registers and clients’ individual patient medical records. The output data was extracted from the MNH registers, and the process data of clinical care elements and complication management were extracted from the individual patient records to measure the required care elements are given and complications are managed according to the national complication protocol. During the data extraction data elements were cross-checked among the registers and individual patient records; if there is a discrepancy, that element was dropped.

In general, input elements were assessed through interviews in 32 health facility heads and department heads; process elements of the quality of MNH care were assessed by using data extracted from a total of 1920 individual patient records in 32 health facilities, and output elements were assessed from 32 health facility MNH registers.

### Data analysis

Data was entered in to an excel database and cleaning was done by running simple frequencies and looking for unusual (out of the coding value) and incomplete values. The selected variables for this study were extracted, coded, and exported to STATA version 13 for analysis.

Elements of the input, process, and outcome variable were coded, analyzed and described using average scores.

To look the difference of care provision among the facility type, average score was used to compare health centers and hospitals.

Further analysis was done based on the operational definition of “satisfactory quality” and “unsatisfactory quality” using the cutoff point of meeting at least 75% of the standards using the previous study [12]. An analysis of the average score of MNH quality of care was done separately for input, process and outcome and then for the overall score. Each element contributes equally to the score for the respective variable.

For the input variable, number of health facilities were used as denominator and number of sample patient records for MNH clinical care process variable and number of complicated cases for the complication management process variable. Estimated number of deliveries were used as denominator for calculating the output standards of antenatal care, skilled births and postnatal care. First antenatal care visit was used as the denominator for syphilis test variable.

### Ethical approval and consent

The data was collected primary for the improvement purpose in the health facilities, which is part of a broader IHI project evaluation study that was reviewed and approved by Ethiopian Public Health Association (EPHA) Scientific and Ethical Review Committee (Ref: EPHA/OG/5046/17). It was purely program evaluation and was waived by the aforementioned IRB in accordance with Ethiopia ethical guideline [13]. Then, a permission was obtained from IHI Ethiopia project office to analyze further the stored data for this manuscript. During the data collection process, informed consent was obtained from all interview subjects and from health facility heads on behalf of mothers for the medical record reviews since not possible to trace them. Confidentiality of the information and their privacy were respected throughout the data collection process and then after. All responses given by the participants have been kept anonymous and confidential using coding system whereby no one has access to the information.

## Results

### Facility characteristics

The study was conducted in a total of 32 health facilities: 5 hospitals and 27 health centers. Twelve health facilities were in Fogera, Amhara, 8 were in Limu Bilbilu, Oromia, 6 were in Tanqua Abergele, Tigray and 6 were in Duguna Fango, SNNPR. A dedicated labour room, maternity ward, operation theatre and neonatal intensive care unit (NICU) were found to be available in all the five hospitals. All except the operation theatre & NICU were also available at the 27 health centers surveyed.

### Input component

The combined mean score for the infrastructure, availability of equipment/supply, and essential drugs of input component of MNH quality was 62% (Table 2).

**Table 2:** Availability of infrastructure, equipment/supply and essential drugs to provide quality MNH care in the facility

Input Quality items	Number of Health facilities that responded “YES”	% Health facilities (#32)
**Infrastructure**		
Functional incinerator on the time of visit.	26	81%
Functional placenta pit on the time of visit.	30	94%
Electric power supplies any type.	30	94%
Water availability in the compound either in the form of pipe water or hand pump.	21	66%
**Mean Infrastructure availability**		**83.6%**
**Health facilities with all infrastructure inputs available**	**9**	**28%**
**Equipment/supply**		
Newborn corner in the delivery room.	10	31%
Baby Weighing scales for Newborns with 50 precision.	31	97%
Complete delivery sets (Kidney dish, forceps, gauze swabs, Scissors, pads, cord tie/clips) at least three per facility.	29	91%
Towel for baby drying and wrapping	4	13%
Bulb suction	23	72%
Ambu bag & face masks (neonatal- size 0)	12	38%
Ambu bag & face masks (neonatal- size 1)	14	44%
Oxygen concentrator/Oxygen cylinder	10	31%
Radiant warmer	15	47%
Running water at the delivery room.	9	34%
Soap at the delivery room.	21	66%
**Mean Equipment/supply availability**		**46%**
**Health facilities with all Equipment/supply inputs available**	**0**	**0**
**Essential drugs**		
• Dexamethasone/betamethasone IV	13	41%
• Methyldopa	23	72%
• Hydralazine	24	75%
• Nifedipine	21	66%
• Ampicillin (IV)	26	81%
• Gentamycin (IV)	30	94%
• Metronidazole (IV)	18	56%
• TTC eye ointment	30	94%
• Vitamin K	28	88%
• MgSO4	23	72%
• Calcium gluconate	10	31%
• Oxytocin	31	97%
• Normal saline/Ringers lactate	32	100%
**Mean of essential drugs availability**		**74%**
**Health facilities with all essential drug inputs available**	**3**	**9%**
**Overall Input Mean**		**62%**

### Infrastructure

Eighty-one percent of the facilities had a functional incinerator and only 66% of the facilities had water available in the compound either in the form of pipe water or hand pump. Less than one third (28%) of the health facilities had all infrastructure inputs available. However, the mean infrastructure availability score was 83.6% (Table 2) which is much higher than equipment/supply and essential drugs availability.

### Equipment & supplies

Only 10 (31%) health facilities had newborn corners in the delivery room. Among the 32 health facilities, only 4 (13%) of them availed towels for drying and wrapping babies in the delivery room. Ninety-one percent of the surveyed health facilities had a complete delivery set in their labour wards. Twelve (38%) and 14 (44%) of them availed artificial manual breathing unit (Ambu) bags and face masks of neonatal sizes 0 and 1, respectively. Oxygen concentrator/oxygen cylinder were availed in 10 (31%) health facilities. Radiant warmers were available in 15 (47%) of the health facilities (Table 2). The mean equipment/supply availability score was 46%, which is low as compared to other input items, infrastructure & essential drugs availability. However, no health facilities had all equipment/supply inputs available at the time of the study.

### Essential drugs

All health facilities availed normal saline in their delivery room. Twenty-three (72%), 10 (31%), and 31 (97%) had Magnesium sulphate (MgSO_4_), calcium gluconate, and oxytocin in the delivery rooms respectively (Table 2). The mean of essential drugs availability score was 74% (Table 2) and only 3 health facilities with all essential drug inputs available.

### Process component

**Fig. 1:**
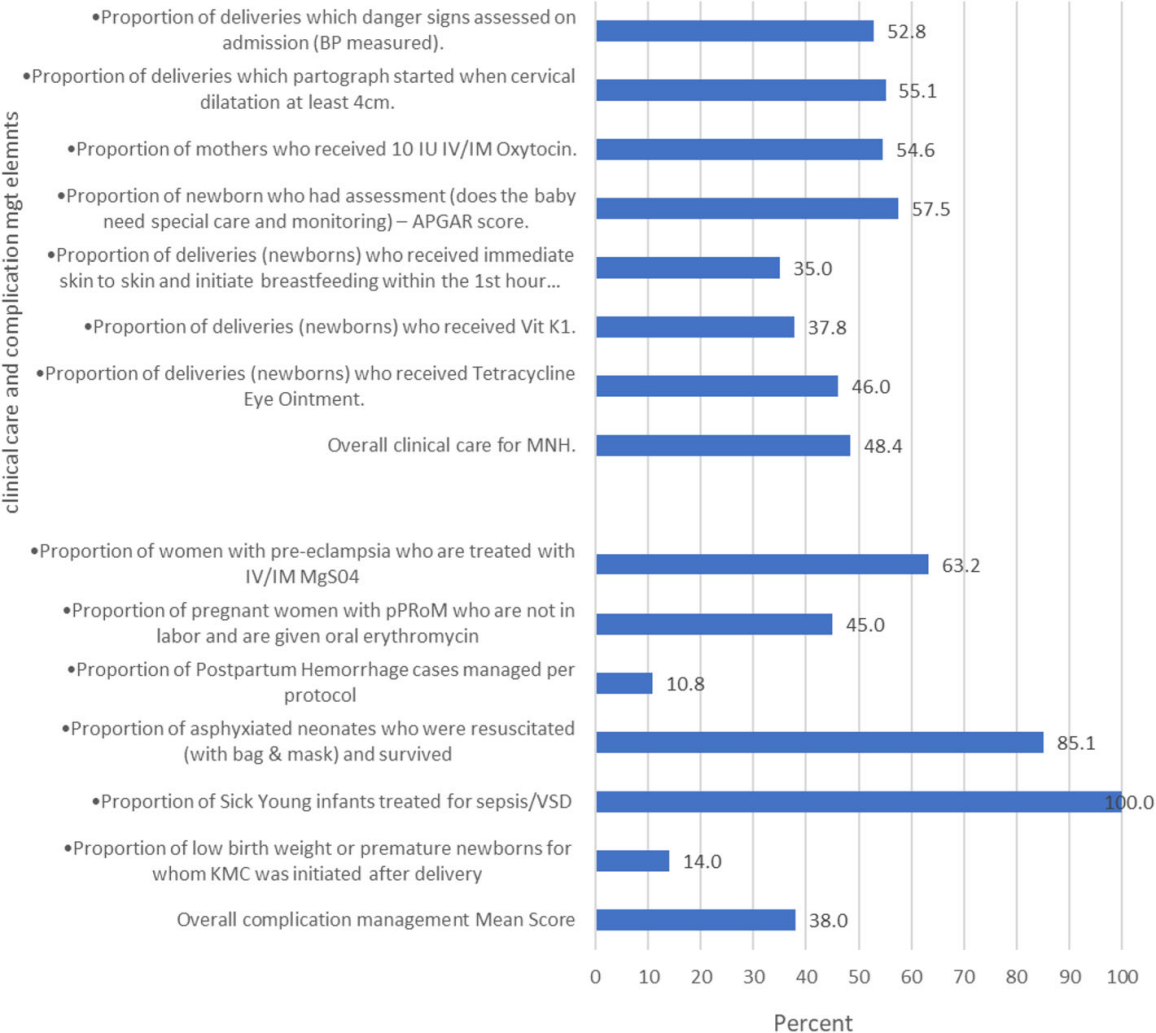
Clinical quality of care process and complication management in MNH care in the health facilities surveyed (number of cases for clinical care =1920)

The mean score for overall clinical care process for MNH was 48% (Fig. 1). Fifty three percent of mothers were assessed for danger signs at admission to the delivery room. Among the 1920 cases, only 38% of newborns received vitamin K, and 35% of newborns had skin-to-skin contact with their mothers and breastfed within 1 h after delivery (Fig. 1). Figure 1 shows that 46% of newborns received Tetracycle (TTC) eye ointment after delivery and 58% of newborns received an assessment to determine if they needed special care and monitoring.

The mean score for overall complication management was 38%. Only 11% of postpartum hemorrhage cases were managed per protocol. Additionally, 45% of pregnant women with preterm premature rupture of the membrane (PPRoM) who were not in labour were given oral erythromycin. This study also revealed that 63% of women with pre-eclampsia were treated with IV/IM MgSO_4_ (Fig. 1).

Though all (100%) sick young infants were treated for sepsis/VSD and most (85%) of the asphyxiated neonates were resuscitated (with bag and mask) and survived, kangaroo mother care (KMC) was initiated only for 14% of low birth weight or premature newborns after delivery (Fig. 1).

### Output

**Fig. 2:**
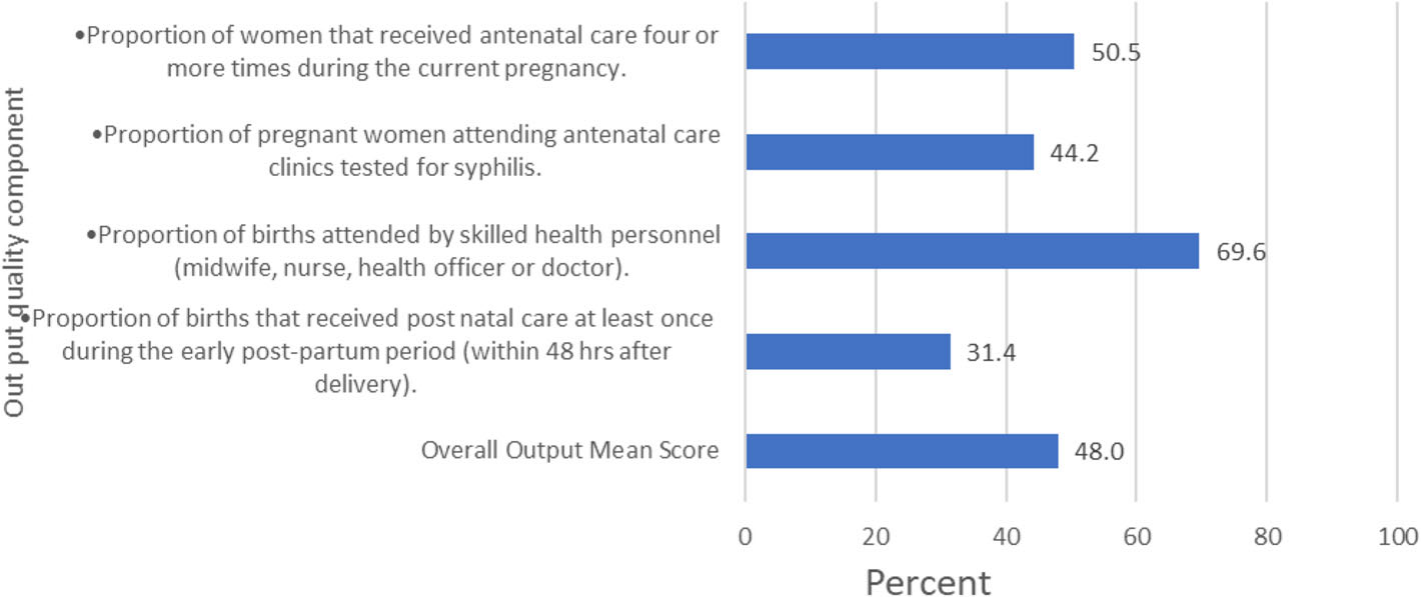
Output quality components in MNH care in the health facility

Seventy percent of births were attended by skilled health personnel (i.e. midwife, nurse, health officer or doctor) while only31% of mothers received postnatal care at least once during the early post-partum period (within 48 h after delivery). The overall outputs mean score was 48% (Fig. 2).

**Fig. 3:**
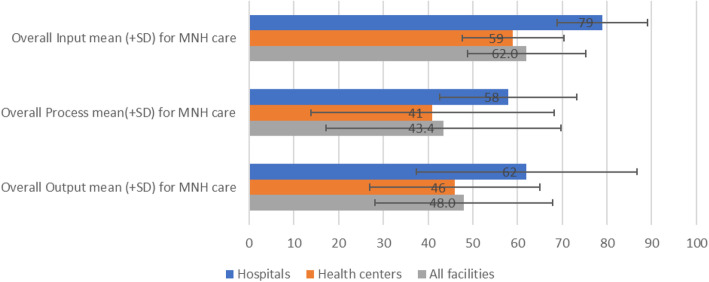
Overall input, process and output components in MNH care in the health facility

This study also analyzed the performance of input, process and output standards by facility type. Overall, hospitals and health centers, respectively, achieved 79 and 59% of the input, 58 and 41% of process, and 62 and 46% of output standards (Fig. 3). No significant difference by facility type.

In this study, analysis was done to determine the proportion of health facilities who met at least 75% of the MNH quality of care standards which were rated as satisfactory. No single health facility met 75% the standards of all the three quality of care components. According to the standard cut-off point for MNH quality of care, only 15.6, 9.3 and 10.7% of health facilities met the satisfactory level in terms of input, process and output components, respectively (Table 3).

**Table 3:** MNH quality of care in health facilities

Quality Component	Health Facility	Number (%) of Health facilities scored > =75% (Satisfactory)	Number (%) of Health facilities scored < 75% (Not Satisfactory)	Total
**Input**	**Health centers**	2 (7.4)	25 (92.6)	27
	**Hospitals**	3 (60)	2 (40)	5
	**All facilities**	5 (15.6)	27 (84.4)	32
**Process**	**Health centers**	3 (11.1)	24 (88.9)	27
	**Hospitals**	0 (0)	5 (100)	5
	**All facilities**	3 (9.4)	29 (90.6)	32
**Output**	**Health centers**	2 (7.7)	24 (92.3)	26
	**Hospitals**	1 (33.3)	2 (66.7)	3
	**All facilities**	3 (10.7)	26 (89.7)	29

## Discussion

This study provides evidence on the performance of selected hospitals and health centers in four regions in Ethiopia against the WHO and HSTQ maternal and newborn health quality of care standards. Most of the health facilities are far from meeting the quality standards. No health facility included in this study met the standards of all three quality of care components. According to the standard cutoff point for MNH quality of care, only 15.6, 9.3 and 10.7% of health facilities met the satisfactory level quality in terms of input, process and output standards, respectively. The result brought the attention of health system leaders and health care providers to intervene. The health system leaders, health care providers and project staffs met together and developed a joint plan to fill the gaps and to improve the service provision. The health care providers in the health facility had used the findings to improve their own gaps through applying quality improvement methodologies. Different supports were provided to the health facilities by the health system leaders and project staffs at multiple levels.

This study has several strengths. The study draws on data from facilities in four most populous agrarian regions of Ethiopia. Using multiple sources of data: interview, chart and register reviews & data collection by senior project staffs, who have prior experience in the subject area were strengths of the study. Moreover, this study addresses broader areas of quality of care as compared to similar previous studies in Ethiopia [14–16].

Although MoH is investing in human resource and infrastructure, the quality of care provided to patients is very poor. When we analyzed the human resources in each health facility, there were a minimum of eight skilled birth attendants (i.e. midwives, nurses, health officers and medical doctors). While investing in the health infrastructure and deploying health cadres, it is important to consider quality of care provision in the health care system. Other studies also shows similar findings [12]. Moreover, evidences shows in addition to accessing to health care, good quality of care is needed to improve outcomes [17–20].

This result revealed that there was a large variation among the quality of care provided by health facilities in each of the three components [16, 21]. Unequitable distribution of support in terms of equipment, supply, supportive supervision and clinical mentorship and the geographical location of the health facility may affect their performance. Another study conducted in Bangladesh, India and other areas found much better performance and the range was minimal among the health facilities [21–23]. Such variation may happen due to the system strength and the attention given to health car provision.

Furthermore, this study finds key elements of inputs that are very necessary in the prevention of maternal and newborn deaths and enhancing the life of mothers and newborns were poorly available [14, 24]. These are issues that need to be addressed and there must be serious consideration on how to address these shortages as it can compromise the quality of maternal and newborn health care and may lead to maternal and newborn morbidity and mortality. Evidence shows poor-quality care is large barrier to reducing maternal and neonatal mortality, and 60% of deaths from conditions amenable to health care are due to poor-quality care [17].

The process component was assessed for the clinical care given and complication management. The clinical care should be given for every mother and newborn and the complication management should be given to mothers and newborns who experienced health complications. This study reveals that overall clinical care provision and complication management were very low. Specifically, PPH complication management which is the major cause of maternal deaths was very low [1]; a study conducted in Kenya shows that PPH complication management is better when we compare it to this study [25]. This difference maybe due to the system support to provide quality of care. Moreover, skin-to-skin contact immediately after birth and breastfeeding initiation within 1 h was very low in this study; whereas, the study done in Kenya shows that a better care of babies that had skin-to-skin contact and experienced breastfeeding within the first hour and babies received vitamin k [14, 25]. Those results mean that health facilities generally provide poor quality care which could be a contributing factor in many maternal and newborn deaths [26].

Maternal and newborn quality of care assessment needs to assess the capacity of health systems as well as the experiences of women [27]. However, we haven’t included women’s experience of care in this assessment, and we recommend future studies to include it to be more comprehensive. Purposive selection of the health facilities may limit representativeness: Health facilities were selected for the purpose of the project. Review of charts instead of direct observation of care may have limited the validity of data: individual patient records were used to collect the clinical care and complication management data. This study also used estimation for denominators of the output quality component and records for the process component as its limitation. Despite those limitations this study determines the quality of MNH care in the health facilities and future research should focus to explore on the reason why the health facilities provide such low level of care for generalization.

## Conclusion

This study revealed that the majority of health facilities did not meet the national MNH quality standards. Focus should be directed more towards improving the process components of the MNH quality of care. Health care providers and program managers need to work to meet the quality standards by designing a strategy that addresses the gap to provide good quality. There also needs to be a quality of MNH study that assesses both the experience of care and provision of care. This study only examined the quality of care being provided. This potential study should include a higher representation of health facilities and include regions that need special support.

## Supplementary Information

**Additional file 1.** English version of the questionnaire entitled as “Baseline Assessment Tool _ HC&Hos _ V4, Abera Biadgo, et”.

**Additional file 2.** Authors title, credentials, email and institution entitles as “Authors information”.

**Additional file 3.**

**Additional file 4.**

**Additional file 5.**

**Additional file 6.**

## Data Availability

Data is available in the supplementary file.

## Abbreviations

ANC: Antenatal care
HSTP: Health sector transformation plan
HSTQ: Health sector transformation in quality
IHI: Institute for Healthcare Improvement
MNH: Maternal and neonatal health
MoH: Ministry of Health
MRN: Medical record numbers
NICU: Neonatal intensive care unit
NQS: National quality strategy
PNC: Postnatal care
QI: Quality improvement
RHB: Regional Health Bureaus
SBA: Skilled birth attendants
WHO: World Health Organization
ZHD: Zonal health departments
