# Supplementary material for: Quality of maternal and newborn health care in Ethiopia: a cross-sectional study
Source: BMC Health Serv Res. 2021 Jul 10;21:679. doi: 10.1186/s12913-021-06680-1 (PMC8272353; doi:10.1186/s12913-021-06680-1)
Supplement: Supplementary file 1 — Additional file 1. English version of the questionnaire entitled as “Baseline Assessment Tool _ HC&Hos _ V4, Abera Biadgo, et”. [file 12913_2021_6680_MOESM1_ESM.pdf]

Health Center and Hospital Baseline Assessment Tool

Section 1. General Information

Name of Facility: ……

Type of Facility: 1. Health Center ☐ 2. Hospital ☐

Region: …………………………………….. Zone: ……………………………… Woreda: ………………………………..

Telephone (landline): ……………………………….

Facility Head Name: ………………………………… Facility head cell phone ………………………………………..

Respondent Name: ………………………………… Respondent position: ………………………………………..

List health facilities (health post, health center) in your catchment:

1.	6.
2.	7.
3.	8.
4.	9.
5.	10.

Population Size:

	2008 EFY	2009EFY
Total Population		
Expected Pregnancies		
Expected deliveries		
Surviving infants		

Date of assessment: ________________________________

Name of Assessors:

1. ____
2. ____
3. ____

Section 2. Staffing

Respondent Name: ……………………………………… Respondent position: ………………………………………..

Total number of staffs		
**Technical Staff**	**No.**	**Remarks**
Specialist doctors		
General practitioners		
IESO		
Anesthetists		
Health officers		
Nurses		
Midwives		
Laboratory personnel		
Pharmacy personnel		
**Supportive staff**		
Data personnel		
Cleaners		
Other supportive staff		
**Total**		

Training topic(s)	Total Trained	Trained L&D or MNCH	Remarks
CEmONC			
BEmONC			
IMNCI			
PMTCT			
HBB			
Comprehensive Post Abortion Care (CPAC)			
Long Acting Family Planning			
Comprehensive Family Planning			
Essential New Born Care (ENBC)			
NICU			
Non-pneumatic Anti-shock Garment (NASG)			
Quality Improvement/Quality Assurance Training			
Data Quality Training			
Other Trainings relevant for MNH:<br>-<br>-<br>-			

Section 3. Patient Feedback Mechanism

Respondent Name: ………………………………….. Respondent position: ……………………………………….

	Yes – 1<br>No - 0	Remarks
Has your facility had a patient satisfaction survey completed in the last 6 months? (please ask the frequency)		
Can you please share the results of patient satisfaction survey or feedback from clients/family/community with us? Document the last 1 year result.<br>NB: Please collect results if can be shared.		
Do you use suggestion box?		
If Yes, how often do you review it?		
Do you have other means of getting feedback from clients, family & community?<br>If yes, describe.		
Is there a Patients' Rights Charter for the facility?		
Visibly placed to guide staff & patients?		

Section 4. Death & near-miss audits

Respondent Name: ………………………………….. Respondent position: ……………………………………….

Do you carry out the following audits in your facility?	Yes – 1<br>No - 0	Remarks
• Maternal death surveillance and response		
• Neonatal death audits?		
• Perinatal death audits?		
• Near-miss audits?		

Section 5. Referral Process

Respondent Name: ………………………………… Respondent position: ………………………………………

	Yes - 1<br>No - 0	Remark
Functional contact numbers for referral centers		
Functional, Well-equipped Ambulance<br>If no, alternative means of patient transport?		
Liaison officer / referral focal person		

Section 6. Existing Quality Improvement (QI) structure and initiatives in the facility

Respondent Name: ………………………………… Respondent position: ………………………………………

	Yes – 1<br>No - 0	Comment
Has there been any QI initiative in this facility in the past 1 year?<br><br>If yes, provide brief detail or list of the initiatives in comment section.		
Is there a QI team in the facility? (If Yes, please list the designations of the team members – use comment section)		
How frequently does the team meet?		
Is there an external organization or unit supporting the QI work in your facility? [If Yes, provide name(s) in comment section]		

Section 7. Supportive supervision

Respondent Name: ………………………………… Respondent position: ………………………………………

	Yes – 1<br>No - 0	Remarks
Is there regular supportive supervision from HC to HP (hospital to HC) for MNH program? (refers to clinical aspect)		
If yes, how frequent it is?		
If yes, have you used MNH program checklist?		
If yes, were feedbacks given? (please check the feedback document)		

Section 8. Review meetings (Please check the minute book)

Respondent Name: ………………………………… Respondent position: ……………………………………….

	Yes - 1<br>No - 0	Remarks
How many monthly PHCU review meetings were conducted at Hospital/PHCU level in 2008EFY? (please put in number)		
Was MNH program discussed?		
Were the HEWs participated?		
Were the health center staff participated? (Hospital)		
Were the minutes taken?		
Was an action plan developed following the performance review?		

Section 9. Data quality

Respondent Name: ………………………………… Respondent position: ……………………………………….

Is there data quality checking system?	Yes -1<br>No - 0	Remarks
If yes, how frequent?		

Section 10. Local Partners working in the Facility

Respondent Name: ………………………………… Respondent position: ……………………………………….

	Name of Organization	Area/Project	Contact details/Remarks
1			
2			
3			
4			
5			

Section 11. Infrastructure and Environment

Respondent Name: ………………………………….. Respondent position: ……………………………………….

Score the level of cleanliness and organization of the following units (1-3 scale – see Operational Definition)

	Scale /score	Remarks
Overall compound		
MCH OPD (ANC)		
Wards (maternity ward)		
Labour/delivery room		
Operation Theatre		
Pharmacy		
Laboratory		
Toilets		

	Yes -1<br>No - 0	Comments:
Facility has functional incinerator		
Facility has functional placenta pit		
Does the facility have electric power supply		
What is the source of power supply?	0. Power Grid/Line<br>1. Generator<br>2. Solar<br>3. Other	
Is there also back-up power supply?		
On average, how many hours is the power supply running? ………………………………..		

What is the source of water?	0. Pipe water<br>1. Hand pump<br>2. Well<br>3. Spring<br>4. River<br>5. Other	
Is there a back water reservoir (tanker) within the facility?	1- Yes<br>0. No	

Section 12. ANC

Respondent Name: ……………………………….. Respondent position: …………………………………….

**Equipment list - ANC**	Number	Remark
Examination couch		
Weighing scales (Adult)		
Blood pressure apparatus		
Stethoscope		
Thermometer		
Fetoscope / Doppler		
Clean Linen for examination coach		
Height measurement board		
Fundal height measurement tape		
Gestation calendar/gestational wheel		
Ultrasound machine		
HIV test kit		

**Protocols:**	**Available Yes – 1 No - 0**	**Utilized Yes – 1 No - 0**	**Remarks**
PMTCT guidelines			
Focused ANC poster			
Infection prevention and control protocol			

Infection Prevention - ANC	**Yes – 1 No - 0**	**Remark**
Is there written infection prevention protocol?		
Running water		
Soap		
Personal towel for drying		
Alcohol hand rub		

Drugs - ANC	Available Yes – 1, No - 0	Stock out in past 3 months (yes – 1, No – 0)	Remarks
Iron			
Folic acid			
Antimalarials			
TT vaccine			
Antiretroviral drugs			
Nevirapine syrup			
Mebendazole			

Section 13. Delivery Unit

Respondent Name: ………………………………… Respondent position: ………………………………………

	Yes – 1<br>No - 0	Remarks
Is there a designated space for KMC?		
Is there Newborn corner in the delivery room?		
Is there NICU in the facility?		
Is there an ambulance which serves the facility?		
Describe the situation (functionality, fuel and adequacy, alternative mechanisms)<br>____<br>____		

**Equipment List - Delivery**	**Number**	**Remarks**
Weighing scales (New born with 50gm precision)		
Admission		
Recovery Beds		
Delivery couch		
Delivery light		
Number of complete delivery sets (Kidney dish, forceps, gauze swabs, Scissors, pads, cord tie/clips)		
Towel for drying and wrapping		
Baby hat		
Number of Vaginal Speculums		
Number of Tear/episiotomy repair sets (Swabs, Cotton, episiotomy scissors, needle holders, drape-toothed/non-toothed forceps)		
Bulb/pigeon suction		
Suction machine		
Ambu bag & face masks (adult)		
Ambu bag & face masks (neonatal- size 0)		
Ambu bag & face masks (neonatal- size 1)		
Heater		
Radiant warmer		
Incubator		

**Equipment list - Delivery**	Number	Remark
Phototherapy light/unit		
Nasal catheter		
Nasogastric tubes size 5-8		
Urethral catheter		
Oxygen concentrator		
Oxygen cylinder		
Respiratory rate timer (Wall watch)		
Tongue depressors (describe type)		
Guedel (oropharyngeal) airway		
Cannulae (different sizes)		
Support Binder for KMC (KMC wrap)		
Feeding cups		
Breast pump		
Blood sugar testing sticks		
Blood specimen bottles		
Urine specimen bottles		
HIV test kit		

**Infection Prevention – Labor & Delivery**	**Yes – 1**<br>**No - 0**	**Remark**
Is there written infection prevention protocol?		
Running water		
Soap		
Personal towel for drying		
Alcohol hand rub		
Eye google		
Apron		
Boots		
Face mask		
Cape		
Elbow length glove (sterile)		
Do they use triple buckle system?<br>o 0.5% chlorine solution for decontamination<br>o Soapy water for rinsing<br>o Clean water further rinsing		

Use three buckets for solid waste segregation (Red, Yellow, Black)		
Safety box for sharp materials		
Autoclave		
Sterilization drums		
Heavy duty gloves for cleaners		
Bin liners (plastic liner)		
Antiseptics		
Brooms, Brushes etc for cleaning equipment		
Sufficient bed linens and theatre drapes		
Demarcated sterilization area for washing & decontamination, packing of instruments and autoclaving.		

**Protocols – Labor and Delivery**	**Available Yes – 1 No – 0**	**Utilized Yes – 1 No – 0**	**Remarks**
Management Protocol on Selected Obstetrics Topics for Health Centers, 2010			
MgSO4 administration protocol			
BEmONC/CEmONC training manual			
Technical and procedural guidelines for safe abortion services in Ethiopia, 2014			
Essential newborn care guideline			
Integrate management of newborn and child Illness			
Newborn resuscitation flow chart (HBB poster)			
Newborn corner guideline			
AMTSL poster			
National newborn case management protocol			
Neonatal intensive care unit guide			
Infection prevention and control protocol			

Section 14. Operating Theatre

Respondent Name: ……………………………….. Respondent position: …………………………………….

**Equipment List - Operating theatre**	Number	Remark
Oxygen concentrator/Oxygen cylinder		
Pulse oxymeters		
Infant resuscitation table		
Suction machine (state Manual or Electric)		
Ambu bag & face masks (adult)		
Ambu bag & face masks neonatal size(0)		
Ambu bag & face mask size(1)		
Adjustable Operating table		
Operating light (UPS back-up?)		
If operating light available, what condition?		
Number of C-section sets		
Number of Laparatomy sets		
Number of hysterectomy sets		
Number of evacuation sets		
Air-conditioner		

Section 15. Pharmacy

Respondent Name: …………………………………… Respondent position: ……………………………………………………

Drug availability - Pharmacy		Available<br>Yes – 1<br>No - 0	Stock out in past 3 months<br>Yes -1, No - 0	Remarks
Dexamethasone/bethamethasone IV				
Hydrocortisone IV				
Analgesics PO				
Analgesics IM				
40% glucose				
**Antihypertensives**	Methyldopa- 1st line			
	Labetalol			
	Hydralazine			
	Nifedipine			
Antidiabetic for GDM (**Insulin)**				
Miconazole Vaginal pessary				
Neomycin + Polymixin B + Nystatin Tablet (vaginal)				
**ANTIBIOTICS**				
Ampicillin (IV)				
Amoxicillin (PO)				
Erythromycin (PO)				
Gentamycin (IV)				
Metronidazole (IV)				
Pen G				
Ceftriaxone				
TTC eye ointment				
**Others**				
Vitamin K				
MgSO4				
Diazepam				
Calcium gluconate				
Oxytocin				
Ergometrine				
Misoprostol				
Pethidine				
Naloxone				
Adrenaline				

**Drug availability - Pharmacy**	**Available**<br>**Yes – 1**<br>**No - 0**	**Stock out in past 3 months**<br>**Yes -1, No - 0**	**Remarks**
Aminophylline			
Salbutamol			
Ketamine			
Lidocaine/Lignocaine			
Atropine			
FLUIDS			
Normal saline			
5% dextrose			
Ringers lactate			
**CONTRACEPTIVES**			
Condoms			
Oral contraceptives			
Emergency contraception			
Hormonal injections			
Implants			
Intrauterine devices			
RAPID Diagnostic Test Kits			
HIV test kits			
Syphilis test kits			

Section 16. Laboratory

Respondent Name: ………………………………….. Respondent position: ……………………………………….

	Yes – 1<br>No - 0	Remarks:
Blood glucose		
Haemoglobin		
Haematocrit (PCV)		
Urine dipstick		
Urine microscopy		
Microscopy for malaria		
VDRL/RPR		
Blood Group		
HIV test Kit		
**Only for hospitals**		
Full blood count		
Liver function tests		
Renal function tests		
Serum electrolytes		
CD4 count		
HIV plasma viral loads		
Blood culture		
Cross match		
Bilirubin		
CSF microscopy		
HBsAg		

Section 17. MNH M&E Tools

Respondent Name: ………………………………… Respondent position: ……………………………………….

Data collection tools	Available<br>Yes – 1<br>No - 0	Utilized<br>Yes – 1<br>No - 0	Remarks
Individual patient folders and sheets			
Registers:			
- ANC			
- Labour & Delivery			
- PNC			
- Family Planning			
- Abortion Care			
- PMTCT			
- Referral			
Tallies Report Formats:			
- ANC Tally Sheet			
- FP Tally Sheet			
- Integrated Maternal & Neonatal Card			
- Monthly Report Format			
- Quarterly Report Format			
- Annual Report Format			
Other Templates:			
- Partograph			
- Cardex /Drug chart			
- Stock card			
- Bin card			

Section 18. HMIS Data + Validation (register review)

HMIS													Validation			
**Indicator**	May16	Juln6	Jul16	Aug16	Sept16	Oct 16	Nov16	Dec16	Jan17	Feb17	Mar17	Apr17	Jan17	Feb17	Mar17	Apr17
Number of pregnant women with at least one ANC visit (H/HC 1.1.2.1)																
Number of pregnant women that received antenatal care at least four visits (H/HC 1.1.3.1)																
Number of pregnant women tested for syphilis (H/HC 1.1.4.1)																
Total number of births attended by skilled health attendants (H/HC C.1.1.5.1)																
Number of postnatal visits within 48 hours of delivery (H/HC 1.1.7.1)																
Number of postnatal visits within 2-3 days of delivery (H/HC 1.1.7.2,)																
Number of postnatal visits within 4-7 days of delivery (H/HC 1.1.7.3,)																
Number of maternal deaths in health facility (H/HC C1.1.1.10.1)																
Number of stillbirths (H/HC/ 1.1.12.1)																
Number of live births (H/HC/ 1.1.12.2)																
Number of babies born in a facility who were pre-term at or under 2500g at birth																
Number of neonatal deaths in the first 24hrs of life (institutional)																
Number of neonatal deaths between 1 and 6 days of life (institutional)																

Delivery Register Review - Complications

**Delivery Register**	**May-16**	**Jun-16**	**Jul-16**	**Aug-16**	**Sep-16**	**Oct-16**	**Nov-16**	**Dec-16**	**Jan 17**	**Feb 17**	**Mar 17**	**Apr 17**
# of pregnant women admitted to a health facility with pre-eclampsia or eclampsia												
# of pregnant women admitted to a health facility with pPRoM												
Number of Postpartum Hemorrhage cases												
Number of neonates with perinatal asphyxia treated with oxygen/resuscitation												
Number of neonates with perinatal asphyxia												
Number of neonates treated for sepsis with antibiotics												
Number of neonates with sepsis												
Number of newborns born <2500g and initiated KMC in the facility												

Section 19. Medical Record Review - Complications

**Chart Audit for complications**	**May-16**	**Jun-16**	**Jul-16**	**Aug-16**	**Sep-16**	**Oct-16**	**Nov-16**	**Dec-16**	**Jan 17**	**Feb 17**	**Mar 17**	**Apr 17**
# of pregnant women admitted to a health facility with pre-eclampsia or eclampsia who are treated with IV/IM MgSO4, per protocol												
# of pregnant women admitted to a health facility with pPRoM and treated with erythromycin or ampicillin, per protocol												
Number of Postpartum Hemorrhage cases managed per protocol												
- Call for help												
- Initiate resuscitation (crystalloids)												
- Oxytocin 10IU IM;												
- Attempt CCT												
- Stimulate contraction by rubbing the uterus												
- Follow up: Check and rub the uterus every 15 minute for 2 hours												
- Monitor vital signs												
- Catheterize and measure urine output												

Section 20. Archived Annual Plan

**Archived Annual Plan**	**May-16**	**Jun-16**	**Jul-16**	**Aug-16**	**Sep-16**	**Oct-16**	**Nov-16**	**Dec-16**	**Jan 17**	**Feb 17**	**Mar 17**	**Apr 17**
Total number of expected deliveries												
Number of expected pregnancies												

Section 21. Medical Record Review – Bundle Month 1: ________________________

(0=Not documented 1=Documented, 3=No space for documenting): use additional sheet to add more medial record review

		MRN	MRN	MRN	MRN	MRN	MRN	MRN	MRN	MRN	MRN	**Total**
On admission	Danger sign assessment (BP measurement)											
	Partograph started when cervical dilatation at least 4cm											
	Availability of soap, water, alcohol hand rub, and gloves											
	Birth companion encouraged to be present during labour and at birth											
	Mothers privacy maintained during labor and delivery											
Before pushing	Availability of gloves, soap/savlon and clean water											
	Preparation of 10 IU IV/IM Oxytocin in syringe (measured by oxytocin administration)											
	Availability of two clean, dry, warm towels and suction device (all elements)											
	Availability of bag and mask (size 0 and 1) both masks)											
	Helper/Assistant identified and informed for resuscitation											
Soon after birth (with 1hr)	Newborn assessment (does the baby need special care and monitoring) – APGAR score											
	Immediate skin to skin and initiate breastfeeding within the 1st hour (measured by mother/baby bonding)											
	Baby weighed and recorded											
	Administer Vit K1 to newborn											
	Administer Tetracycline Eye Ointment to newborn											
	**Total**											

Medical Record Review – Bundle   Month 2: ________________________

(0=Not documented 1=Documented, 3=No space for documenting): use additional sheet to add more medial record review

		MRN	MRN	MRN	MRN	MRN	MRN	MRN	MRN	MRN	MRN	**Total**
On admission	Danger sign assessment (BP measurement)											
	Partograph started when cervical dilatation at least 4cm											
	Availability of soap, water, alcohol hand rub, and gloves											
	Birth companion encouraged to be present during labour and at birth											
	Mothers privacy maintained during labor and delivery											
Before pushing	Availability of gloves, soap/savlon and clean water											
	Preparation of 10 IU IV/IM Oxytocin in syringe (measured by oxytocin administration)											
	Availability of two clean, dry, warm towels and suction device (all elements)											
	Availability of bag and mask (size 0 and 1) both masks)											
	Helper/Assistant identified and informed for resuscitation											
Soon after birth (with 1hr)	Newborn assessment (does the baby need special care and monitoring) – APGAR score											
	Immediate skin to skin and initiate breastfeeding within the 1st hour (measured by mother/baby bonding)											
	Baby weighed and recorded											
	Administer Vit K1 to newborn											
	Administer Tetracycline Eye Ointment to newborn											
	**Total**											

Medical Record Review – Bundle Month 3: ______________________

(0=Not documented 1=Documented, 3=No space for documenting): use additional sheet to add more medial record review

		MRN	MRN	MRN	MRN	MRN	MRN	MRN	MRN	MRN	MRN	**Total**
On admission	Danger sign assessment (BP measurement)											
	Partograph started when cervical dilatation at least 4cm											
	Availability of soap, water, alcohol hand rub, and gloves											
	Birth companion encouraged to be present during labour and at birth											
	Mothers privacy maintained during labor and delivery											
Before pushing	Availability of gloves, soap/savlon and clean water											
	Preparation of 10 IU IV/IM Oxytocin in syringe (measured by oxytocin administration)											
	Availability of two clean, dry, warm towels and suction device (all elements)											
	Availability of bag and mask (size 0 and 1) both masks)											
	Helper/Assistant identified and informed for resuscitation											
Soon after birth (with 1hr)	Newborn assessment (does the baby need special care and monitoring) – APGAR score											
	Immediate skin to skin and initiate breastfeeding within the 1st hour (measured by mother/baby bonding)											
	Baby weighed and recorded											
	Administer Vit K1 to newborn											
	Administer Tetracycline Eye Ointment to newborn											
	**Total**											

Medical Record Review – Bundle   Month 4: ____________________

(0=Not documented 1=Documented, 3=No space for documenting): use additional sheet to add more medial record review

		MRN	MRN	MRN	MRN	MRN	MRN	MRN	MRN	MRN	MRN	**Total**
On admission	Danger sign assessment (BP measurement)											
	Partograph started when cervical dilatation at least 4cm											
	Availability of soap, water, alcohol hand rub, and gloves											
	Birth companion encouraged to be present during labour and at birth											
	Mothers privacy maintained during labor and delivery											
Before pushing	Availability of gloves, soap/savlon and clean water											
	Preparation of 10 IU IV/IM Oxytocin in syringe (measured by oxytocin administration)											
	Availability of two clean, dry, warm towels and suction device (all elements)											
	Availability of bag and mask (size 0 and 1) both masks)											
	Helper/Assistant identified and informed for resuscitation											
Soon after birth (with 1hr)	Newborn assessment (does the baby need special care and monitoring) – APGAR score											
	Immediate skin to skin and initiate breastfeeding within the 1st hour (measured by mother/baby bonding)											
	Baby weighed and recorded											
	Administer Vit K1 to newborn											
	Administer Tetracycline Eye Ointment to newborn											
	**Total**											

Medical Record Review – Bundle Month 5: ________________________

(0=Not documented 1=Documented, 3=No space for documenting): use additional sheet to add more medial record review

		MRN	MRN	MRN	MRN	MRN	MRN	MRN	MRN	MRN	MRN	**Total**
On admission	Danger sign assessment (BP measurement)											
	Partograph started when cervical dilatation at least 4cm											
	Availability of soap, water, alcohol hand rub, and gloves											
	Birth companion encouraged to be present during labour and at birth											
	Mothers privacy maintained during labor and delivery											
Before pushing	Availability of gloves, soap/savlon and clean water											
	Preparation of 10 IU IV/IM Oxytocin in syringe (measured by oxytocin administration)											
	Availability of two clean, dry, warm towels and suction device (all elements)											
	Availability of bag and mask (size 0 and 1) both masks)											
	Helper/Assistant identified and informed for resuscitation											
Soon after birth (with 1hr)	Newborn assessment (does the baby need special care and monitoring) – APGAR score											
	Immediate skin to skin and initiate breastfeeding within the 1st hour (measured by mother/baby bonding)											
	Baby weighed and recorded											
	Administer Vit K1 to newborn											
	Administer Tetracycline Eye Ointment to newborn											
	**Total**											

Medical Record Review – Bundle Month 6: ______________________

(0=Not documented 1=Documented, 3=No space for documenting): use additional sheet to add more medial record review

		MRN	MRN	MRN	MRN	MRN	MRN	MRN	MRN	MRN	MRN	**Total**
On admission	Danger sign assessment (BP measurement)											
	Partograph started when cervical dilatation at least 4cm											
	Availability of soap, water, alcohol hand rub, and gloves											
	Birth companion encouraged to be present during labour and at birth											
	Mothers privacy maintained during labor and delivery											
Before pushing	Availability of gloves, soap/savlon and clean water											
	Preparation of 10 IU IV/IM Oxytocin in syringe (measured by oxytocin administration)											
	Availability of two clean, dry, warm towels and suction device (all elements)											
	Availability of bag and mask (size 0 and 1) both masks)											
	Helper/Assistant identified and informed for resuscitation											
Soon after birth (with 1hr)	Newborn assessment (does the baby need special care and monitoring) – APGAR score											
	Immediate skin to skin and initiate breastfeeding within the 1st hour (measured by mother/baby bonding)											
	Baby weighed and recorded											
	Administer Vit K1 to newborn											
	Administer Tetracycline Eye Ointment to newborn											
	**Total**											
