# Supplementary material for: Quality of maternal and newborn health care in Ethiopia: a cross-sectional study
Source: BMC Health Serv Res. 2021 Jul 10;21:679. doi: 10.1186/s12913-021-06680-1 (PMC8272353; doi:10.1186/s12913-021-06680-1)
Supplement: Supplementary file 2 — Additional file 2. Authors title, credentials, email and institution entitles as “Authors information”. [file 12913_2021_6680_MOESM2_ESM.docx]

**Quality of maternal and newborn health care in Ethiopia: A cross-sectional study**

| Name | Title | credentials | Email | Author’s institution |
| --- | --- | --- | --- | --- |
| Abera Biadgo | Mr | MPH | abiadgo@ihi.org/ a.b.kefale@gmail.com | Institute for Healthcare Improvement, Ethiopia Project Office |
| Aynalem Legesse | Ms | MSc | aynalemalegesse@gmail.com | Ministry of health of Ethiopia, Addis Ababa Ethiopia |
| Abiy Seifu Estifanos | Mr | MPH | seifu9@gmail.com | Department of Reproductive, Family and Population Health, School of Public Health, College of Health Sciences, Addis Ababa University |
| Kavita Singh | Dr | Ph.D | singhk@email.unc.edu | Department of Maternal and Child Health, Gillings School of Global Public Health, University of North Carolina at Chapel Hill, 135 Dauer Dr, Chapel Hill, NC 27599, USA |
| Zewdie Mulissa | Dr | MD | zmulissa@yahoo.com | Institute for Healthcare Improvement, Ethiopia Project Office |
| Hema Magge | Dr | MD | Hema.Magge@gatesfoundation.org | Brigham and Women’s Hospital Division of Global Health Equity (Boston, MA) |
| Abiyou Kiflie | Dr | MD | akiflie@IHI.org | Institute for Healthcare Improvement, Ethiopia Project Office |
| Befikadu Bitewulign | Mr | MPH | bbitewulign@ihi.org | Institute for Healthcare Improvement, Ethiopia Project Office |
| Mehiret Abate | Mrs | MPH | mabate@ihi.org | Institute for Healthcare Improvement, Ethiopia Project Office |
| Haregeweyni Alemu | Mrs | MPH | halemu@IHI.org | Institute for Healthcare Improvement, Ethiopia Project Office |

Authors information
