# Supplementary material for: Quality of maternal and newborn health care in Ethiopia: a cross-sectional study
Source: BMC Health Serv Res. 2021 Jul 10;21:679. doi: 10.1186/s12913-021-06680-1 (PMC8272353; doi:10.1186/s12913-021-06680-1)
Supplement: Supplementary file 6 — Additional file 6. [file 12913_2021_6680_MOESM6_ESM.docx]

use "C:\Users\user\Desktop\2. MNH Quality care level_Jan 24, 2019.dta"

codebook Facilityhasfunctionalincinerat

codebook Facilityhasfunctionalplacenta

codebook Doesthefacilityhaveelectricp Whatisthesourceofpowersuppl Istherealsobackuppowersuppl

codebook Doesthefacilityhaveelectricp

codebook Istherealsobackuppowersuppl

recode Istherealsobackuppowersuppl (0=0) (1/2=1),gen (powerbackupsupply)

codebook powerbackupsupply

codebook Whatisthesourceofwater

recode Whatisthesourceofwater (0=1) (1/5=0),gen (wateravaliblity)

list Whatisthesourceofwater wateravaliblity

tab Isthereabackwaterreservoir

tab IsthereadesignatedspaceforK

tab IsthereNewborncornerinthede

gen InputInfrastructure= powerbackupsupply+ wateravaliblity+ Facilityhasfunctionalincinerat+ Facilityhasfunctionalplacenta+ Doesthefacilityhaveelectricp+ Isthereabackwaterreservoir+ IsthereadesignatedspaceforK+ IsthereNewborncornerinthede

tab InputInfrastructure

label variable InputInfrastructure "Input for infrastruction score"

tab Examinationcouch

recode Examinationcouch (0=0) (1/max=1), gen (ExamCoachANC)

list Examinationcouch ExamCoachANC

tab WeighingscalesAdult

recode WeighingscalesAdult (0=0) (1/max=1), gen (WeightScaleAdult)

tab Bloodpressureapparatus

recode Bloodpressureapparatus (0=0) (1/max=1), gen (BloodpressureapparatusNew)

tab Stethoscope

recode Stethoscope (0=0) (1/max=1), gen (StethoscopeANC)

tab Thermometer

recode Thermometer (0=0) (1/max=1), gen (ThermometerANC)

tab FetoscopeDoppler

recode FetoscopeDoppler (0=0) (1/max=1), gen (FetoscopeDopplerANC)

tab CleanLinenforexaminationcoach

recode CleanLinenforexaminationcoach (0=0) (1/max=1), gen (CleanLinenANC)

tab Heightmeasurementboard

tab Fundalheightmeasurementtape

recode Fundalheightmeasurementtape (0=0) (1/2=1), gen (FundalheighttapeANC)

tab Gestationcalendargestationalwh

recode Gestationcalendargestationalwh (0=0) (1/2=1), gen (GestationcalendarANC)

tab Ultrasoundmachine

gen InputANCmaterials= Heightmeasurementboard+ Ultrasoundmachine+ ExamCoachANC+ WeightScaleAdult+ BloodpressureapparatusNew+ StethoscopeANC+ ThermometerANC+ FetoscopeDopplerANC+ CleanLinenANC+ FundalheighttapeANC+ GestationcalendarANC

tab InputANCmaterials

label variable InputANCmaterials "ANC examination supply and equipments score"

tab Admissionbed

recode Admissionbed (0/2=0) (3/8=1), gen (AdmissionbedLabor)

tab RecoveryBeds

recode RecoveryBeds (0/2=0) (3/6=1), gen (PNCBeds)

tab Deliverycouch

recode Deliverycouch (0/1=0) (2/6=1), gen (DeliverycouchNew)

tab Deliverylight

recode Deliverylight (0=0) (1/4=1), gen (DeliverylightNew)

tab Numberofcompletedeliverysets

recode Numberofcompletedeliverysets (0/2=0) (3/12=1), gen (deliverySets)

tab Towelfordryingandwrapping

recode Towelfordryingandwrapping (0=0) (1/max=1), gen (TowelDryingWrapping)

tab Babyhat

tab NumberofVaginalSpeculums

recode NumberofVaginalSpeculums (0/1=0) (2/12=1), gen (VaginalSpeculums)

tab NumberofTearepisiotomyrepair

recode NumberofTearepisiotomyrepair (0/1=0) (2/11=1), gen (episiotomySet)

tab Bulbpigeonsuction

recode Bulbpigeonsuction (0/1=0) (2/6=1), gen (BulbpigeonsuctionNew)

tab Suctionmachine

recode Suctionmachine (0=0) (1/3=1), gen (SuctionmachineNew)

tab Ambubagfacemasksadult

recode Ambubagfacemasksadult (0=0) (1/10=1), gen (mbubagmasksadult)

tab Ambubagfacemasksneonatal

recode Ambubagfacemasksneonatal (0=0) (1/9=1), gen (facemasksneonatal0)

tab FE

recode FE (0=0) (1/8=1), gen (facemaskNeonatal1)

tab Heater

recode Heater (0=0) (1/3=1), gen (Heater_room)

tab Radiantwarmer

recode Radiantwarmer (0=0) (1/3=1), gen (RadiantwarmerNew)

tab Oxygenconcentrator

tab Oxygenconcentrator

save "C:\Users\user\Desktop\2. MNH Quality care level_Jan 24, 2019.dta", replace

tab Oxygencylinder

gen O2concentratororCylinder=.

replace O2concentratororCylinder=0 if Oxygenconcentrator==0 & Oxygencylinder==0

replace O2concentratororCylinder=1 if O2concentratororCylinder==.

list Oxygenconcentrator Oxygencylinder O2concentratororCylinder

save "C:\Users\user\Desktop\2. MNH Quality care level_Jan 24, 2019.dta", replace

save "C:\Users\user\Desktop\2. MNH Quality care level_Jan 24, 2019.dta", replace

tab RespiratoryratetimerWallwatc

recode RespiratoryratetimerWallwatc (0=0) (1/2=1), gen(timerWallwatch)

tab Cannulaedifferentsizes

gen CannulaedifferentsizesNew=.

replace CannulaedifferentsizesNew=0 if Cannulaedifferentsizes=="0"

replace CannulaedifferentsizesNew=1 if CannulaedifferentsizesNew==.

tab SupportBinderforKMCKMCwrap

recode SupportBinderforKMCKMCwrap (0=0) (1/6=1), gen (BinderforKMCKMCwrap)

tab Feedingcups

tab Bloodsugartestingsticks

tab Bloodspecimenbottles

tab FY

recode FY (0=0) (1/max=1)

recode FY (0=0) (1/max=1), gen (HIVtestkitN)

tab WeighingscalesNewbornwith50

recode WeighingscalesNewbornwith50 (0=0) (1/4=1), gen ( WeighingNewbornwith50)

save "C:\Users\user\Desktop\2. MNH Quality care level_Jan 24, 2019.dta", replace

gen InputDeliverySupply= Babyhat+ Bloodsugartestingsticks+ Bloodspecimenbottles+ AdmissionbedLabor+ PNCBeds+ DeliverycouchNew+ DeliverylightNew+ deliverySets+ TowelDryingWrapping+ VaginalSpeculums+ episiotomySet+ BulbpigeonsuctionNew+ SuctionmachineNew+ mbubagmasksadult+ facemasksneonatal0+ facemaskNeonatal1+ Heater_room+ RadiantwarmerNew+ O2concentratororCylinder+ timerWallwatch+ CannulaedifferentsizesNew+ BinderforKMCKMCwrap+ HIVtestkitN+ WeighingNewbornwith50

label variable InputDeliverySupply "Inputs for delivery supplies and equipements"

save "C:\Users\user\Desktop\2. MNH Quality care level_Jan 24, 2019.dta", replace

label variable InputDeliverySupply "Inputs for delivery supplies and equipements score"

tab FZ

tab GA

tab GB

tab GC

tab GD

tab1 Eyegoogle Apron Boots Facemask Cape Elbowlengthglovesterile Dotheyusetriplebucklesystem Usethreebucketsforsolidwaste Safetyboxforsharpmaterials Autoclave Sterilizationdrums Heavydutyglovesforcleaners Binlinersplasticliner Antiseptics BroomsBrushesetcforcleaning Sufficientbedlinensandtheatre Demarcatedsterilizationareafor

recode Apron (0=0) (1/max=1), gen (ApronN)

recode Boots (0=0) (1/max=1), gen ( BootsN)

recode Facemask (0=0) (1/max=1), gen (FacemaskN)

gen bucketsTriple=.

replace bucketsTriple=1 if Dotheyusetriplebucklesystem==1

replace bucketsTriple=0 if bucketsTriple==.

save "C:\Users\user\Desktop\2. MNH Quality care level_Jan 24, 2019.dta", replace

gen AutoclaveN=.

replace AutoclaveN=1 if Autoclave=="1"

replace AutoclaveN=0 if AutoclaveN==.

tab Binlinersplasticliner

tab Sufficientbedlinensandtheatre

gen InfectionPreventionScore= FZ+ GA+ GB+ GC+ GD+ Eyegoogle+ Cape+ Elbowlengthglovesterile+ Dotheyusetriplebucklesystem+ Safetyboxforsharpmaterials+ Sterilizationdrums+ Heavydutyglovesforcleaners+ Antiseptics+ BroomsBrushesetcforcleaning+ Binlinersplasticliner+ Demarcatedsterilizationareafor+ ApronN+ BootsN+ FacemaskN+ bucketsTriple+ AutoclaveN

label variable InfectionPreventionScore "Infection prevention score at delivery room"

tab1 ManagementProtocolonSelectedO GW MgSO4administrationprotocolava MgSO4administrationprotocoluti BEmONCCEmONCtrainingmanualava BEmONCCEmONCtrainingmanualuti Technicalandproceduralguidelin HC Essentialnewborncareguideline HE Integratemanagementofnewborna HG Newbornresuscitationflowchart HI Newborncornerguidelineavailabl Newborncornerguidelineutilized AMTSLposteravailable AMTSLposterutilized Nationalnewborncasemanagement HO

gen InfectionPreventionScoreN= FZ+ GA+ GB+ GC+ GD+ Eyegoogle+ Cape+ Elbowlengthglovesterile+ Sterilizationdrums+ Heavydutyglovesforcleaners+ Antiseptics+ BroomsBrushesetcforcleaning+ Binlinersplasticliner+ Demarcatedsterilizationareafor+ ApronN+ BootsN+ FacemaskN+ AutoclaveN

label variable InfectionPreventionScoreN "Infection prevention score at delivery room_input"

drop InfectionPreventionScore

gen managmentprotocolAvailibility= ManagementProtocolonSelectedO+ MgSO4administrationprotocolava+ BEmONCCEmONCtrainingmanualava+ Technicalandproceduralguidelin+ Essentialnewborncareguideline+ Integratemanagementofnewborna+ Newbornresuscitationflowchart+ Newborncornerguidelineavailabl+ AMTSLposteravailable+ Nationalnewborncasemanagement

label variable managmentprotocolAvailibility "MNH managment protocol score"

gen managmentprotocolUtilized= GW+ MgSO4administrationprotocoluti+ BEmONCCEmONCtrainingmanualuti+ HC+ HE+ HG+ HI+ Newborncornerguidelineutilized+ AMTSLposterutilized+ HO

label variable managmentprotocolUtilized "MNH managment protocol utilization score"

label variable managmentprotocolAvailibility "MNH managment protocol availability score"

gen IPutilization= Dotheyusetriplebucklesystem+ Safetyboxforsharpmaterials+ bucketsTriple

drop IPutilization

gen IPutilization= Dotheyusetriplebucklesystem+ Safetyboxforsharpmaterials+ bucketsTriple+ DR

label variable IPutilization "Infection prevention utilization score for four elements"

label variable IPutilization "Infection prevention practice score for four elements"

s

gen Inputscore= InputInfrastructure+ InputANCmaterials+ InputDeliverySupply+ InfectionPreventionScoreN+ managmentprotocolAvailibility

tab Inputscore

tab1 InputInfrastructure InputANCmaterials InputDeliverySupply InfectionPreventionScoreN managmentprotocolAvailibility

gen InputPercentage= Inputscore/75

tab InputPercentage

drop InputPercentage

gen InputPercentage= (Inputscore/75)*100

tab InputPercentage

gen InputStandard=.

replace InputStandard = 1 if InputPercentage>=80

replace InputStandard = 0 if InputPercentage<80

label variable InputStandard "Input variables meeting standards"

label define InputStandard 1 "Good input quality" 0 "Not good input quality"

label value InputStandard InputStandard

tab InputStandard

tab1 BulbpigeonsuctionNew SuctionmachineNew mbubagmasksadult facemasksneonatal0 facemaskNeonatal1 Heater_room RadiantwarmerNew

use "C:\Users\user\Desktop\2. MNH Quality care level_Jan 24, 2019.dta"

codebook Facilityhasfunctionalincinerat

codebook Facilityhasfunctionalplacenta

codebook Doesthefacilityhaveelectricp Whatisthesourceofpowersuppl Istherealsobackuppowersuppl

codebook Doesthefacilityhaveelectricp

codebook Istherealsobackuppowersuppl

recode Istherealsobackuppowersuppl (0=0) (1/2=1),gen (powerbackupsupply)

codebook powerbackupsupply

codebook Whatisthesourceofwater

recode Whatisthesourceofwater (0=1) (1/5=0),gen (wateravaliblity)

list Whatisthesourceofwater wateravaliblity

tab Isthereabackwaterreservoir

tab IsthereadesignatedspaceforK

tab IsthereNewborncornerinthede

gen InputInfrastructure= powerbackupsupply+ wateravaliblity+ Facilityhasfunctionalincinerat+ Facilityhasfunctionalplacenta+ Doesthefacilityhaveelectricp+ Isthereabackwaterreservoir+ IsthereadesignatedspaceforK+ IsthereNewborncornerinthede

tab InputInfrastructure

label variable InputInfrastructure "Input for infrastruction score"

tab Examinationcouch

recode Examinationcouch (0=0) (1/max=1), gen (ExamCoachANC)

list Examinationcouch ExamCoachANC

tab WeighingscalesAdult

recode WeighingscalesAdult (0=0) (1/max=1), gen (WeightScaleAdult)

tab Bloodpressureapparatus

recode Bloodpressureapparatus (0=0) (1/max=1), gen (BloodpressureapparatusNew)

tab Stethoscope

recode Stethoscope (0=0) (1/max=1), gen (StethoscopeANC)

tab Thermometer

recode Thermometer (0=0) (1/max=1), gen (ThermometerANC)

tab FetoscopeDoppler

recode FetoscopeDoppler (0=0) (1/max=1), gen (FetoscopeDopplerANC)

tab CleanLinenforexaminationcoach

recode CleanLinenforexaminationcoach (0=0) (1/max=1), gen (CleanLinenANC)

tab Heightmeasurementboard

tab Fundalheightmeasurementtape

recode Fundalheightmeasurementtape (0=0) (1/2=1), gen (FundalheighttapeANC)

tab Gestationcalendargestationalwh

recode Gestationcalendargestationalwh (0=0) (1/2=1), gen (GestationcalendarANC)

tab Ultrasoundmachine

gen InputANCmaterials= Heightmeasurementboard+ Ultrasoundmachine+ ExamCoachANC+ WeightScaleAdult+ BloodpressureapparatusNew+ StethoscopeANC+ ThermometerANC+ FetoscopeDopplerANC+ CleanLinenANC+ FundalheighttapeANC+ GestationcalendarANC

tab InputANCmaterials

label variable InputANCmaterials "ANC examination supply and equipments score"

tab Admissionbed

recode Admissionbed (0/2=0) (3/8=1), gen (AdmissionbedLabor)

tab RecoveryBeds

recode RecoveryBeds (0/2=0) (3/6=1), gen (PNCBeds)

tab Deliverycouch

recode Deliverycouch (0/1=0) (2/6=1), gen (DeliverycouchNew)

tab Deliverylight

recode Deliverylight (0=0) (1/4=1), gen (DeliverylightNew)

tab Numberofcompletedeliverysets

recode Numberofcompletedeliverysets (0/2=0) (3/12=1), gen (deliverySets)

tab Towelfordryingandwrapping

recode Towelfordryingandwrapping (0=0) (1/max=1), gen (TowelDryingWrapping)

tab Babyhat

tab NumberofVaginalSpeculums

recode NumberofVaginalSpeculums (0/1=0) (2/12=1), gen (VaginalSpeculums)

tab NumberofTearepisiotomyrepair

recode NumberofTearepisiotomyrepair (0/1=0) (2/11=1), gen (episiotomySet)

tab Bulbpigeonsuction

recode Bulbpigeonsuction (0/1=0) (2/6=1), gen (BulbpigeonsuctionNew)

tab Suctionmachine

recode Suctionmachine (0=0) (1/3=1), gen (SuctionmachineNew)

tab Ambubagfacemasksadult

recode Ambubagfacemasksadult (0=0) (1/10=1), gen (mbubagmasksadult)

tab Ambubagfacemasksneonatal

recode Ambubagfacemasksneonatal (0=0) (1/9=1), gen (facemasksneonatal0)

tab FE

recode FE (0=0) (1/8=1), gen (facemaskNeonatal1)

tab Heater

recode Heater (0=0) (1/3=1), gen (Heater_room)

tab Radiantwarmer

recode Radiantwarmer (0=0) (1/3=1), gen (RadiantwarmerNew)

tab Oxygenconcentrator

tab Oxygenconcentrator

save "C:\Users\user\Desktop\2. MNH Quality care level_Jan 24, 2019.dta", replace

tab Oxygencylinder

gen O2concentratororCylinder=.

replace O2concentratororCylinder=0 if Oxygenconcentrator==0 & Oxygencylinder==0

replace O2concentratororCylinder=1 if O2concentratororCylinder==.

list Oxygenconcentrator Oxygencylinder O2concentratororCylinder

save "C:\Users\user\Desktop\2. MNH Quality care level_Jan 24, 2019.dta", replace

save "C:\Users\user\Desktop\2. MNH Quality care level_Jan 24, 2019.dta", replace

tab RespiratoryratetimerWallwatc

recode RespiratoryratetimerWallwatc (0=0) (1/2=1), gen(timerWallwatch)

tab Cannulaedifferentsizes

gen CannulaedifferentsizesNew=.

replace CannulaedifferentsizesNew=0 if Cannulaedifferentsizes=="0"

replace CannulaedifferentsizesNew=1 if CannulaedifferentsizesNew==.

tab SupportBinderforKMCKMCwrap

recode SupportBinderforKMCKMCwrap (0=0) (1/6=1), gen (BinderforKMCKMCwrap)

tab Feedingcups

tab Bloodsugartestingsticks

tab Bloodspecimenbottles

tab FY

recode FY (0=0) (1/max=1)

recode FY (0=0) (1/max=1), gen (HIVtestkitN)

tab WeighingscalesNewbornwith50

recode WeighingscalesNewbornwith50 (0=0) (1/4=1), gen ( WeighingNewbornwith50)

save "C:\Users\user\Desktop\2. MNH Quality care level_Jan 24, 2019.dta", replace

gen InputDeliverySupply= Babyhat+ Bloodsugartestingsticks+ Bloodspecimenbottles+ AdmissionbedLabor+ PNCBeds+ DeliverycouchNew+ DeliverylightNew+ deliverySets+ TowelDryingWrapping+ VaginalSpeculums+ episiotomySet+ BulbpigeonsuctionNew+ SuctionmachineNew+ mbubagmasksadult+ facemasksneonatal0+ facemaskNeonatal1+ Heater_room+ RadiantwarmerNew+ O2concentratororCylinder+ timerWallwatch+ CannulaedifferentsizesNew+ BinderforKMCKMCwrap+ HIVtestkitN+ WeighingNewbornwith50

label variable InputDeliverySupply "Inputs for delivery supplies and equipements"

save "C:\Users\user\Desktop\2. MNH Quality care level_Jan 24, 2019.dta", replace

label variable InputDeliverySupply "Inputs for delivery supplies and equipements score"

tab FZ

tab GA

tab GB

tab GC

tab GD

tab1 Eyegoogle Apron Boots Facemask Cape Elbowlengthglovesterile Dotheyusetriplebucklesystem Usethreebucketsforsolidwaste Safetyboxforsharpmaterials Autoclave Sterilizationdrums Heavydutyglovesforcleaners Binlinersplasticliner Antiseptics BroomsBrushesetcforcleaning Sufficientbedlinensandtheatre Demarcatedsterilizationareafor

recode Apron (0=0) (1/max=1), gen (ApronN)

recode Boots (0=0) (1/max=1), gen ( BootsN)

recode Facemask (0=0) (1/max=1), gen (FacemaskN)

gen bucketsTriple=.

replace bucketsTriple=1 if Dotheyusetriplebucklesystem==1

replace bucketsTriple=0 if bucketsTriple==.

save "C:\Users\user\Desktop\2. MNH Quality care level_Jan 24, 2019.dta", replace

gen AutoclaveN=.

replace AutoclaveN=1 if Autoclave=="1"

replace AutoclaveN=0 if AutoclaveN==.

tab Binlinersplasticliner

tab Sufficientbedlinensandtheatre

gen InfectionPreventionScore= FZ+ GA+ GB+ GC+ GD+ Eyegoogle+ Cape+ Elbowlengthglovesterile+ Dotheyusetriplebucklesystem+ Safetyboxforsharpmaterials+ Sterilizationdrums+ Heavydutyglovesforcleaners+ Antiseptics+ BroomsBrushesetcforcleaning+ Binlinersplasticliner+ Demarcatedsterilizationareafor+ ApronN+ BootsN+ FacemaskN+ bucketsTriple+ AutoclaveN

label variable InfectionPreventionScore "Infection prevention score at delivery room"

tab1 ManagementProtocolonSelectedO GW MgSO4administrationprotocolava MgSO4administrationprotocoluti BEmONCCEmONCtrainingmanualava BEmONCCEmONCtrainingmanualuti Technicalandproceduralguidelin HC Essentialnewborncareguideline HE Integratemanagementofnewborna HG Newbornresuscitationflowchart HI Newborncornerguidelineavailabl Newborncornerguidelineutilized AMTSLposteravailable AMTSLposterutilized Nationalnewborncasemanagement HO

gen InfectionPreventionScoreN= FZ+ GA+ GB+ GC+ GD+ Eyegoogle+ Cape+ Elbowlengthglovesterile+ Sterilizationdrums+ Heavydutyglovesforcleaners+ Antiseptics+ BroomsBrushesetcforcleaning+ Binlinersplasticliner+ Demarcatedsterilizationareafor+ ApronN+ BootsN+ FacemaskN+ AutoclaveN

label variable InfectionPreventionScoreN "Infection prevention score at delivery room_input"

drop InfectionPreventionScore

gen managmentprotocolAvailibility= ManagementProtocolonSelectedO+ MgSO4administrationprotocolava+ BEmONCCEmONCtrainingmanualava+ Technicalandproceduralguidelin+ Essentialnewborncareguideline+ Integratemanagementofnewborna+ Newbornresuscitationflowchart+ Newborncornerguidelineavailabl+ AMTSLposteravailable+ Nationalnewborncasemanagement

label variable managmentprotocolAvailibility "MNH managment protocol score"

gen managmentprotocolUtilized= GW+ MgSO4administrationprotocoluti+ BEmONCCEmONCtrainingmanualuti+ HC+ HE+ HG+ HI+ Newborncornerguidelineutilized+ AMTSLposterutilized+ HO

label variable managmentprotocolUtilized "MNH managment protocol utilization score"

label variable managmentprotocolAvailibility "MNH managment protocol availability score"

gen IPutilization= Dotheyusetriplebucklesystem+ Safetyboxforsharpmaterials+ bucketsTriple

drop IPutilization

gen IPutilization= Dotheyusetriplebucklesystem+ Safetyboxforsharpmaterials+ bucketsTriple+ DR

label variable IPutilization "Infection prevention utilization score for four elements"

label variable IPutilization "Infection prevention practice score for four elements"

s

gen Inputscore= InputInfrastructure+ InputANCmaterials+ InputDeliverySupply+ InfectionPreventionScoreN+ managmentprotocolAvailibility

tab Inputscore

tab1 InputInfrastructure InputANCmaterials InputDeliverySupply InfectionPreventionScoreN managmentprotocolAvailibility

gen InputPercentage= Inputscore/75

tab InputPercentage

drop InputPercentage

gen InputPercentage= (Inputscore/75)*100

tab InputPercentage

gen InputStandard=.

replace InputStandard = 1 if InputPercentage>=80

replace InputStandard = 0 if InputPercentage<80

label variable InputStandard "Input variables meeting standards"

label define InputStandard 1 "Good input quality" 0 "Not good input quality"

label value InputStandard InputStandard

tab InputStandard

tab1 BulbpigeonsuctionNew SuctionmachineNew mbubagmasksadult facemasksneonatal0 facemaskNeonatal1 Heater_room RadiantwarmerNew

alpha AKB AKC AKD AKE AKF AKG AKH AKI AKJ AKK AKL

Test scale = mean(unstandardized items)

Average interitem covariance: .3607404

Number of items in the scale: 11

Scale reliability coefficient: 0.7738

tab TypeofFacility

drop if TypeofFacility==0

drop if TypeofFacility==3

drop if TypeofFacility==.

save "C:\Users\user\Desktop\4. MNH Quality care_Jan 28, 2018.dta", replace

tab1 Region Woreda FacilityName TypeofFacility Listhealthfacilitieshealthpo

replace Woreda ="Fogera" if Woreda =="Woreta town"

tab Woreda

tab1 TotalPopulation2008EC ExpectedPregnancies2008EC

tab1 TotalPopulation2009EC ExpectedPregnancies2009EC

tab1 Specialistdoctors Generalpractitioners IESO Anesthetists Healthofficers Nurses Midwives Healthextensionworkers Laboratorypersonnel Pharmacypersonnel Datapersonnel Cleaners Othersupportivestaff Total

tab1 TotaltrainedonCEmONC CEmONCtrainedandworkingatLD TotaltrainedonBEmONC BEmONCtrainedandworkingatLD TotaltrainedonIMNCI IMNCItrainedandworkingatLD TotaltrainedonPMTCT PMTCTtrainedandworkingatLD TotaltrainedHBB HBBtrainedandworkingatLDor TotaltrainedonComprehensivePo ComprehensivePostAbortionCare TotaltrainedonLongActingFami LongActingFamilyPlanningLAFP TotaltrainedonComprehensiveFa ComprehensiveFamilyPlanningCFP TotaltrainedonEssentialNewBo EssentialNewBornCareENBCtra TotaltrainedonNICU NICUtrainedandworkingatLDo TotaltrainedonNonpneumaticAn NonpneumaticAntishockGarment TotaltrainedonQualityImprovem QualityImprovementQualityAssur TotaltrainedonDataQuality DataQualitytrainedandworking Hasyourfacilityhadapatients

tab1 Doyouusesuggestionbox IfYeshowoftendoyoureviewi Doyouhaveothermeansofgettin Ifyesdescribe IsthereaPatientsRightsChart Visiblyplacedtoguidestaffp Maternaldeathsurveillanceandr Neonataldeathaudits Perinataldeathaudits Nearmissaudits Functionalcontactnumbersforre FunctionalWellequippedAmbulan Liaisonofficerreferralfocal

tab1 HastherebeenanyQIinitiative Ifyesprovidebriefdetailorl IsthereaQIteaminthefacilit IfYespleaselistthedesignati Howfrequentlydoestheteammeet Isthereanexternalorganization IfYesprovidenamesincommen Isthereregularsupportivesuper Ifyeshowfrequentitis IfyeshaveyouusedMNHprogram Ifyeswerefeedbacksgiven HowmanymonthlyPHCUreviewmeet WasMNHprogramdiscussed WeretheHEWsparticipated WerethehealthcenterHospital Weretheminutestaken Wasanactionplandevelopedfoll Istheredataqualitycheckingsy Ifyeshowfrequent

tab1 CompoundcleanlinessLevelofc ANCOPDcleanlinessLevelofcl maternitywardcleanlinessLevel Labourdeliveryroomcleanliness OperationTheatrecleanliness PharmacycleanlinessLevelofc LaboratorycleanlinessLevelof ToiletscleanlinessLevelofcl

drop OperationTheatrecleanliness

tab1 Facilityhasfunctionalincinerat Facilityhasfunctionalplacenta Doesthefacilityhaveelectricp Whatisthesourceofpowersuppl Istherealsobackuppowersuppl howmanyhoursisthepowersuppl Whatisthesourceofwater Isthereabackwaterreservoir Examinationcouch WeighingscalesAdult Bloodpressureapparatus Stethoscope Thermometer FetoscopeDoppler CleanLinenforexaminationcoach Heightmeasurementboard Fundalheightmeasurementtape Gestationcalendargestationalwh Ultrasoundmachine HIVtestkit PMTCTguidelinesavailable PMTCTguidelinesutilized FocusedANCposteravailable FocusedANCposterutilized Infectionpreventionandcontrol UseofInfectionpreventionandc Istherewritteninfectionpreven Runningwater Soap Personaltowelfordrying Alcoholhandrub IronAvailable IronStockoutinpast3months FolicacidAvailable FolicacidStockoutinpast3mo AntimalarialsAvailable AntimalarialsStockoutinpast3 TTvaccineAvailable TTvaccineStockoutinpast3mo AntiretroviraldrugsAvailable AntiretroviraldrugsStockoutin NevirapinesyrupAvailable NevirapinesyrupStockoutinpas MebendazoleAvailable MebendazoleStockoutinpast3m IsthereadesignatedspaceforK IsthereNewborncornerinthede IsthereNICUinthefacility Isthereanambulancewhichserve Ifyesdescribefunctionalityf

destring Numberofpregnantwomenwithat, gen (anc1july15)

tab Numberofpregnantwomenwithat

drop Numberofpregnantwomenwithat

destring MY , gen (anc1jul15)

rename anc1july15 anc1jun15

list MY anc1jul15

drop MY

destring MZ , gen (anc1aug15)

drop MZ

destring NA, gen (anc1sep15)

drop NA

rename anc1sep15 anc1oct15

rename anc1aug15 anc1sep15

rename anc1jul15 anc1aug15

rename anc1jun15 anc1jul15

destring NB, gen (anc1Nov15)

drop NB

destring NC , gen (anc1dec15)

drop NC

destring ND, gen (anc1jan16)

drop ND

destring NE, gen (anc1feb16)

drop NE

destring NF, gen (anc1mar16)

drop NF

destring NG, gen (anc1apr16)

drop NG

save "C:\Users\user\Desktop\9. MNH Quality care_Feb13, 2018.dta", replace

destring NH, gen (anc1may16)

destring NI, gen (anc1jun16)

destring NI, gen (anc1jul16)

destring NI, gen (anc1jun16)

drop NH

rename NI anc1jun16

rename NJ anc1jul16

rename NK anc1aug16

gen anc1total14= anc1jul15+ anc1aug15+ anc1sep15+ anc1oct15+ anc1Nov15+ anc1dec15+ anc1jan16+ anc1feb16+ anc1mar16+ anc1apr16+ anc1may16+ anc1jun16+ anc1jul16+ anc1aug16

label variable anc1total14 "Total ANC1 for 14 months"

gen anc1total12= anc1sep15+ anc1oct15+ anc1Nov15+ anc1dec15+ anc1jan16+ anc1feb16+ anc1mar16+ anc1apr16+ anc1may16+ anc1jun16+ anc1jul16+ anc1aug16

label variable anc1total12 "Total ANC1 for 12 months from sep 2015 to Aug 2016 & May 16 to apr 2017 for Fogera"

drop anc1total14

destring Numberofpregnantwomenthatrec, gen (anc4Jul15)

destring NQ, gen (anc4aug15)

destring NR , gen (anc4sep15)

destring NS, gen (anc4oct15)

destring NT, gen (anc4nov15)

destring NU , gen (anc4dec15)

destring NV , gen (anc4jan15)

rename anc4jan15 anc4jan16

destring NW, gen (anc4feb16)

destring NX, gen (anc4mar16)

destring NY , gen (anc4apr16)

destring NZ, gen (anc4may16)

destring OA, gen (anc4jun16)

drop Numberofpregnantwomenthatrec NQ NR NS NT NU NV NW NX NY NZ

rename OA anc4jun16

rename OB anc4july16

rename OC anc4aug16

gen anc4total12= anc4sep15+ anc4oct15+ anc4nov15+ anc4dec15+ anc4jan16+ anc4feb16+ anc4mar16+ anc4apr16+ anc4may16+ anc4jun16+ anc4july16+ anc4aug16

label variable anc4total12 "Total ANC4 for 12 months from sep 2015 to Aug 2016 & May 16 to apr 2017 for Fogera"

save "C:\Users\user\Desktop\9. MNH Quality care_Feb13, 2018.dta", replace

destring OI, gen (syphilis4aug15)

destring OJ , gen (syphilis4sep15)

gen syphilistotal12= syphilis4sep15 + OK + OL+ OM+ ON+ OO+ OP+ OQ+ OR+ OS+ OT+ OU

drop OJ

label variable syphilistotal12 "Total Syphilis for 12 months from sep 2015 to Aug 2016 & May 16 to apr 2017 for Fogera"

destring PB , gen (sbasep15)

drop PB

save "C:\Users\user\Desktop\9. MNH Quality care_Feb13, 2018.dta", replace

gen skilledBA12= sbasep15+ PC+ PD+ PE+ PF+ PG+ PH+ PI+ PJ+ PK+ PL+ PM

label variable skilledBA12 "Total Skilled Birth Attendants for 12 months from sep 2015 to Aug 2016 & May 16 to apr 2017 for Fogera"

gen pnc48hrs12= PT+ PU+ PV+ PW+ PX+ PY+ PZ+ QA+ QB+ QC+ QD+ QE

gen maternalDeath12= QL+ QM+ QN+ QO+ QP+ QQ+ QR+ QS+ QT+ QU+ QV+ QW

gen stillbirth12= RD+ RE+ RF+ RG+ RH+ RI+ RJ+ RK+ RL+ RM+ RN+ RO

gen Livebirth12= RV+ RW+ RX+ RY+ RZ+ SA+ SB+ SC+ SD+ SE+ SF+ SG

gen LBWpreterm12= SN+ SO+ SP+ SQ+ SR+ SS+ ST+ SU+ SV+ SW+ SX+ SY

gen neonataldeath24hrs12= TF+ TG+ TH+ TI+ TJ+ TK+ TL+ TM+ TN+ TO+ TP+ TQ

gen neonataldeath6days12= TX+ TY+ TZ+ UA+ UB+ UC+ UD+ UE+ UF+ UG+ UH+ UI

gen preeclampsia12= UP+ UQ+ UR+ US+ UT+ UU+ UV+ UW+ UX+ UY+ UZ+ VA

gen PROM12= VD+ VE+ VF+ VG+ VH+ VI+ VJ+ VK+ VL+ VM+ VN+ VO

gen PPH12= VR+ VS+ VT+ VU+ VV+ VW+ VX+ VY+ VZ+ WA+ WB+ WC

gen AsphyxiaTx12= WF+ WG+ WH+ WI+ WJ+ WK+ WL+ WM+ WN+ WO+ WP+ WQ

gen AsphyxiaCases12= WT+ WU+ WV+ WW+ WX+ WY+ WZ+ XA+ XB+ XC+ XD+ XE

gen SepsisTx12= XH+ XI+ XJ+ XK+ XL+ XM+ XN+ XO+ XP+ XQ+ XR+ XS

gen SepsisCases12= Numberofneonateswithsepsiss+ Numberofneonateswithsepsiso+ Numberofneonateswithsepsisn+ Numberofneonateswithsepsisd+ Numberofneonateswithsepsisj+ Numberofneonateswithsepsisf+ Numberofneonateswithsepsism+ YC+ YD+ YE+ YF+ YG

gen KMC12= YJ+ YK+ YL+ YM+ YN+ YO+ YP+ YQ+ YR+ YS+ YT+ YU

gen preeclampsiaTx12= YX+ YY+ YZ+ ZA+ ZB+ ZC+ ZD+ ZE+ ZF+ ZG+ ZH+ ZI

gen PROMTx12= ZL+ ZM+ ZN+ ZO+ ZP+ ZQ+ ZR+ ZS+ ZT+ ZU+ ZV+ ZW

gen PPHmgt12= ZZ+ AAA+ AAB+ AAC+ AAD+ AAE+ AAF+ AAG+ AAH++ AAJ+ AAK

gen PPHmgt12= ZZ+ AAA+ AAB+ AAC+ AAD+ AAE+ AAF+ AAG+ AAH+ AAJ+ AAK

gen ExpectedDelivery12= AAN+ AAO+ AAP+ AAQ+ AAR+ AAS+ AAT+ AAU+ AAV+ AAW+ AAX+ AAY

save "C:\Users\user\Desktop\9. MNH Quality care_Feb13, 2018.dta", replace

label variable pnc48hrs12 "Total PNC 48hrs for 12 months from sep 2015 to Aug 2016 & May 16 to apr 2017 for Fogera"

label variable maternalDeath12 "Total Maternal deaths for 12 months from sep 2015 to Aug 2016 & May 16 to apr 2017 for Fogera"

label variable stillbirth12 "Total Still births for 12 months from sep 2015 to Aug 2016 & May 16 to apr 2017 for Fogera"

label variable Livebirth12 "Total Live births for 12 months from sep 2015 to Aug 2016 & May 16 to apr 2017 for Fogera"

label variable LBWpreterm12 "Total LBW and pre-maturity for 12 months from sep 2015 to Aug 2016 & May 16 to apr 2017 for Fogera"

label variable neonataldeath24hrs12 "Total neonatal death within 24hrs for 12 months from sep 2015 to Aug 2016 & May 16 to apr 2017 for Fogera"

label variable neonataldeath6days12 "Total neonatal death 1-6 days for 12 months from sep 2015 to Aug 2016 & May 16 to apr 2017 for Fogera"

label variable preeclampsia12 "Total pre-eclampsia/eclampsia for 12 months from sep 2015 to Aug 2016 & May 16 to apr 2017 for Fogera"

label variable preeclampsia12 "Total pre-eclampsia/eclampsia cases for 12 months from sep 2015 to Aug 2016 & May 16 to apr 2017 for Fogera"

label variable PROM12 "Total PROM cases for 12 months from sep 2015 to Aug 2016 & May 16 to apr 2017 for Fogera"

label variable PPH12 "Total PPH cases for 12 months from sep 2015 to Aug 2016 & May 16 to apr 2017 for Fogera"

label variable AsphyxiaTx12 "Total Asphyxia treatement for 12 months from sep 2015 to Aug 2016 & May 16 to apr 2017 for Fogera"

label variable AsphyxiaCases12 "Total Asphyxia cases for 12 months from sep 2015 to Aug 2016 & May 16 to apr 2017 for Fogera"

label variable SepsisTx12 "Total Sepsis treatement for 12 months from sep 2015 to Aug 2016 & May 16 to apr 2017 for Fogera"

label variable SepsisCases12 "Total Sepsis cases for 12 months from sep 2015 to Aug 2016 & May 16 to apr 2017 for Fogera"

label variable KMC12 "Total KMC for 12 months from sep 2015 to Aug 2016 & May 16 to apr 2017 for Fogera"

label variable preeclampsiaTx12 "Total pre-eclampsia/eclampsia managment for 12 months from sep 2015 to Aug 2016 & May 16 to apr 2017 for Fogera"

label variable PROMTx12 "Total PPROM managment for 12 months from sep 2015 to Aug 2016 & May 16 to apr 2017 for Fogera"

label variable PPHmgt12 "Total PPH managment for 12 months from sep 2015 to Aug 2016 & May 16 to apr 2017 for Fogera"

label variable ExpectedDelivery12 "Total Expected delivery for 12 months from sep 2015 to Aug 2016 & May 16 to apr 2017 for Fogera"

gen PartographTotal6= Partographstartedwhencervical+ ABR+ ACI+ ACZ+ ADQ+ AEH

gen OxytocinTotal6= Preparationof10IUIVIMuterot+ ABS+ ACJ+ ADA+ ADR+ AEI

gen APGAR= Newbornassessmenttotalmonth1+ Newbornassessmenttotalmonth2+ Newbornassessmenttotalmonth3+ Newbornassessmenttotalmonth4+ Newbornassessmenttotalmonth5+ Newbornassessmenttotalmonth6

gen skinToSkinTotal6= Immediateskintoskinandinitia+ ABU+ ACL+ ADC+ ADT+ AEK

gen VitK= AdministerVitK1tonewborntota+ ABV+ ACM+ ADD+ ADU+ AEL

gen TTC= AdministerTetracyclineEyeOintm+ ABW+ ACN+ ADE+ ADV+ AEM

rename APGARtotal6

rename APGAR APGARtotal6

rename VitK VitKtotal6

rename TTC

rename TTC TTCtotal6

label variable PartographTotal6 "Partograph initiation for last six months"

label variable OxytocinTotal6 "Oxytocine adminstration for last six months"

label variable APGARtotal6 "Newborn assessement (APGAR) for last six months"

label variable skinToSkinTotal6 "Immediate skin-to-skin contact for last six months"

label variable VitKtotal6 "Vit K adminstration for last six months"

label variable TTCtotal6 "TTC eye ointment for the new born for last six months"

save "C:\Users\user\Desktop\9. MNH Quality care_Feb13, 2018.dta",

gen anc4proportion= (anc4total12/ ExpectedDelivery12)

tab anc4proportion

gen SBAproportion= (skilledBA12 / ExpectedDelivery12)

tab SBAproportion

gen SyphilisProportion= (syphilistotal12/ anc1total12)

tab SyphilisProportion

gen PNC48hrsProportion= (pnc48hrs12/ ExpectedDelivery12)

tab PNC48hrsProportion

tab1 maternalDeath12 stillbirth12 Livebirth12 LBWpreterm12 neonataldeath24hrs12 neonataldeath6days12 preeclampsia12 PROM12 PPH12 AsphyxiaTx12 AsphyxiaCases12 SepsisTx12 SepsisCases12 KMC12 preeclampsiaTx12 PROMTx12 PPHmgt12 ExpectedDelivery12 PartographTotal6 OxytocinTotal6 APGARtotal6 skinToSkinTotal6 VitKtotal6 TTCtotal6

gen MMR= (maternalDeath12/ Livebirth12*100000)

gen StillBirth= (stillbirth12 / Livebirth12*1000)

gen NMR24hrs= (neonataldeath24hrs12 / Livebirth12*1000)

gen NMR6days= ( neonataldeath6days12 / Livebirth12*1000)

gen PreEclampsiaMgt= ( preeclampsiaTx12 / preeclampsia12)

gen PROMmgt= ( PROMTx12 / PROM12 )

gen PPHmgt= ( PPHmgt12/ PPH12 )

gen AsphyxiaMgt= ( AsphyxiaTx12 / AsphyxiaCases12 )

gen SepsisMgt= ( SepsisTx12/ SepsisCases12 )

gen KMC= ( KMC12/ LBWpreterm12 )

save "C:\Users\user\Desktop\10. MNH Quality care_Feb13, 2018.dta", replace

tab DangerBPpercentage

codebook DangerBPpercentage

recode DangerBPpercentage, gen (DangerSignBPproportion)

gen DangerSignBPproportion=.

tab DangerSignBPproportion

replace DangerSignBPproportion== DangerBPpercentage

replace DangerSignBPproportion= DangerBPpercentage

list DangerBPpercentage DangerSignBPproportion

gen PartographIntiationProportion= (PartographTotal6/60)

gen OxytocinProportion= ( OxytocinTotal6 /60)

gen APGARproportion= ( APGARtotal6 /60)

gen skinToSkinProportion= ( skinToSkinTotal6 /60)

gen VitKproportion= ( VitKtotal6 /60)

gen TTCproportion= ( TTCtotal6 /60)

save "C:\Users\user\Desktop\10. MNH Quality care_Feb13, 2018.dta", replace

list TTCtotal6 TTCproportion

label variable anc4proportion "Proportion of women that received antenatal care four or more times during the current pregnancy"

label variable SBAproportion "Proportion of births attended by skilled health personnel (midwife, nurse, health officer or doctor)"

label variable SyphilisProportion "Proportion of pregnant women attending antenatal care clinics tested for syphilis"

label variable PNC48hrsProportion "Proportion of births that received post natal care at least once during the early post-partum period (within 48 hrs after delivery)"

label variable MMR "Maternal mortality ratio"

label variable StillBirth "Still Birth rate"

label variable NMR24hrs "Neonatal death with in 24 hrs rate per 1000 LB"

label variable StillBirth "Still Birth rate per 1000 LB"

label variable MMR "Maternal mortality ratio per 100000 LB"

label variable NMR6days "Neonatal death 1-6 days rate per 1000 LB"

label variable PreEclampsiaMgt "Proportion of women with pre-eclampsia who are treated with IV/IM MgS04"

label variable PROMmgt "Proportion of pregnant women with pPRoM who are not in labor and are given oral erythromycin"

label variable PPHmgt "Proportion of Postpartum Hemorrhage cases managed per protocol"

label variable AsphyxiaMgt "Proportion of asphyxiated neonates who were resuscitated (with bag & mask) and survived"

label variable SepsisMgt "Proportion of Sick Young infants treated for sepsis/VSD"

label variable KMC "Proportion of low birth weight or premature newborns for whom KMC was initiated after delivery"

label variable DangerSignBPproportion "Proportion of deliveries which danger signs assessed on admission (BP measured)"

label variable PartographIntiationProportion "Proportion of deliveries which partograph started when cervical dilatation at least 4cm"

label variable OxytocinProportion "Proportion of mothers who received 10 IU IV/IM Oxytocin"

label variable APGARproportion "Proportion of newborn who had assessment (does the baby need special care and monitoring) – APGAR score"

label variable skinToSkinProportion "Proportion of deliveries (newborns) who received immediate skin to skin and initiate breastfeeding within the 1st hour (measured by mother/baby bonding)"

label variable VitKproportion "Proportion of deliveries (newborns) who received Vit K1"

label variable TTCproportion "Proportion of deliveries (newborns) who received Tetracycline Eye Ointment"

drop Healthextensionworkers

drop Specialistdoctors Generalpractitioners IESO Anesthetists

drop Datapersonnel Cleaners

drop Admissionbed RecoveryBeds Deliverycouch Deliverylight

drop NumberofVaginalSpeculums

drop FU Boots Sterilizationdrums Autoclave PenGavailable FT FU

drop anc1jul15 anc1aug15 anc1sep15 anc1oct15 anc1Nov15 anc1dec15 anc1jan16 anc1feb16 anc1mar16 anc1apr16 anc1may16 anc1jun16 anc1jul16 anc1aug16 anc4Jul15 anc4aug15 anc4sep15 anc4oct15 anc4nov15 anc4dec15 anc4jan16 anc4feb16 anc4mar16 anc4apr16 anc4may16 anc4jun16 anc4july16 anc4aug16

drop Numberofpregnantwomentestedf OI syphilis4sep15 OK OL OM ON OO OP OQ OR OS OT OU Totalnumberofbirthsattendedb PA sbasep15 PC PD PE PF PG PH PI PJ PK PL PM

drop Numberofpostnatalvisitswithin PS PT PU PV PW PX PY PZ QA QB QC QD QE Numberofmaternaldeathsinheal QK QL QM QN QO QP QQ QR QS QT QU QV QW

drop NumberofstillbirthsHHC11 RC RD RE RF RG RH RI RJ RK RL RM RN RO NumberoflivebirthsHHC11 RU RV RW RX RY RZ SA SB SC SD SE SF SG

drop Numberofbabiesborninafacili SM SN SO SP SQ SR SS ST SU SV SW SX SY Numberofneonataldeathsinthe TE TF TG TH TI TJ TK TL TM TN TO TP TQ

drop Numberofneonataldeathsbetween TW TX TY TZ UA UB UC UD UE UF UG UH UI ofpregnantwomenadmittedtoa UO UP UQ UR US UT UU UV UW UX UY UZ VA

drop VB VC VD VE VF VG VH VI VJ VK VL VM VN VO NumberofPostpartumHemorrhagec VQ VR VS VT VU VV VW VX VY VZ WA WB WC

drop Numberofneonateswithperinatal WE WF WG WH WI WJ WK WL WM WN WO WP WQ WR WS WT WU WV WW WX WY WZ XA XB XC XD XE

drop Numberofneonatestreatedforse XG XH XI XJ XK XL XM XN XO XP XQ XR XS NumberofneonateswithsepsisJ Numberofneonateswithsepsisa Numberofneonateswithsepsiss Numberofneonateswithsepsiso Numberofneonateswithsepsisn Numberofneonateswithsepsisd Numberofneonateswithsepsisj Numberofneonateswithsepsisf Numberofneonateswithsepsism YC YD YE YF YG

drop Numberofnewbornsborn2500gan YI YJ YK YL YM YN YO YP YQ YR YS YT YU YV YW YX YY YZ ZA ZB ZC ZD ZE ZF ZG ZH ZI

drop ZJ ZK ZL ZM ZN ZO ZP ZQ ZR ZS ZT ZU ZV ZW ZX ZY ZZ AAA AAB AAC AAD AAE AAF AAG AAH AAI AAJ AAK

drop Totalnumberofexpecteddeliveri AAM AAN AAO AAP AAQ AAR AAS AAT AAU AAV AAW AAX AAY

drop BPmeasuredonadmissiontotalmo Partographstartedwhencervical Preparationof10IUIVIMuterot Newbornassessmenttotalmonth1 Immediateskintoskinandinitia AdministerVitK1tonewborntota AdministerTetracyclineEyeOintm ABQ ABR ABS Newbornassessmenttotalmonth2 ABU ABV ABW ACH ACI ACJ Newbornassessmenttotalmonth3 ACL ACM ACN ACY ACZ ADA Newbornassessmenttotalmonth4 ADC ADD ADE ADP ADQ ADR Newbornassessmenttotalmonth5 ADT ADU ADV AEG AEH AEI Newbornassessmenttotalmonth6 AEK AEL AEM

summarize Pharmacypersonnel

summarize Pharmacypersonnel

graph bar (mean) TypeofFacility

graph bar (mean) TypeofFacility

tab1 Facilityhasfunctionalincinerat Facilityhasfunctionalplacenta Doesthefacilityhaveelectricp Whatisthesourceofpowersuppl

tab Whatisthesourceofwater

gen WaterAvailability=.

replace WaterAvailability=1 if Whatisthesourceofwater==(0/1)

replace WaterAvailability=0 if Whatisthesourceofwater==(4/5)

list Whatisthesourceofwater WaterAvailability

replace WaterAvailability=1 if Whatisthesourceofwater==0

replace WaterAvailability=1 if Whatisthesourceofwater==1

replace WaterAvailability=0 if Whatisthesourceofwater==4

replace WaterAvailability=0 if Whatisthesourceofwater==5

list Whatisthesourceofwater WaterAvailability

tab WaterAvailability

label variable WaterAvailability "Water availability in the HF either Pipe water or Hand pump"

des WaterAvailability

tab WaterAvailability

sum WaterAvailability

sum DexamethasonebethamethasoneIVa HydrocortisoneIVavailable

sum Methyldopa1stlineavailable Hydralazineavailable Nifedipineavailable AmpicillinIVavailable GentamycinIVavailable MetronidazoleIVavailable TTCeyeointmentavailable VitaminKavailable MgSO4available Calciumgluconateavailable Oxytocinavailable

drop HydrocortisoneIVavailable

drop Misoprostolavailable

gen NormalsalineRL= Normalsalineavailable+ Ringerslactateavailable

tab NormalsalineRL

list NormalsalineRL Normalsalineavailable Ringerslactateavailable

tab1 Normalsalineavailable Ringerslactateavailable

label variable NormalsalineRL "Normalsaline or Ringer Lactate availability"

tab1 DexamethasonebethamethasoneIVa Methyldopa1stlineavailable Hydralazineavailable Nifedipineavailable AmpicillinIVavailable GentamycinIVavailable MetronidazoleIVavailable TTCeyeointmentavailable VitaminKavailable MgSO4available Calciumgluconateavailable Oxytocinavailable

save "C:\Users\user\Desktop\14. MNH Quality care_Feb14, 2018.dta", replace

gen OxygenConcentratorOxygenCylinder= Oxygenconcentrator+ Oxygencylinder

label variable OxygenConcentratorOxygenCylinder "Oxygen concentrator/Oxygen cylinder avalaiblity"

list OxygenConcentratorOxygenCylinder Oxygenconcentrator Oxygencylinder

tab OxygenConcentratorOxygenCylinder

recode OxygenConcentratorOxygenCylinder (0=0) (1/max=1)

tab OxygenConcentratorOxygenCylinder

sum WeighingscalesNewbornwith50 Numberofcompletedeliverysets Towelfordryingandwrapping Bulbpigeonsuction Ambubagfacemasksneonatal EZ Radiantwarmer FV FW

rename EZ Mask1

rename FV RunningWater

rename FW Soap

sum WeighingscalesNewbornwith50 Numberofcompletedeliverysets Towelfordryingandwrapping Bulbpigeonsuction Ambubagfacemasksneonatal Mask1 Radiantwarmer RunningWater Soap

tab1 WeighingscalesNewbornwith50 Numberofcompletedeliverysets Towelfordryingandwrapping Bulbpigeonsuction Ambubagfacemasksneonatal Mask1 Radiantwarmer RunningWater Soap

tab IsthereNewborncornerinthede

recode IsthereNewborncornerinthede (0=0) (1=1), gen (NewbornCorner)

list NewbornCorner IsthereNewborncornerinthede

tab NewbornCorner

tab Isthereanambulancewhichserve

tab Ifyesdescribefunctionalityf

gen AmbulanceWellEquiped=.

replace AmbulanceWellEquiped=1 if Ifyesdescribefunctionalityf== "Good status and available fuel sufficiently"

replace AmbulanceWellEquiped=1 if Ifyesdescribefunctionalityf== "Good status and available fuel sufficiently; Use motor bicycle to inform"

replace AmbulanceWellEquiped=1 if Ifyesdescribefunctionalityf=="Good status and available fuel sufficiently; Use motor bicycle to inform"

replace AmbulanceWellEquiped=1 if Ifyesdescribefunctionalityf=="Good status and available fuel sufficiently; Use motor bicycle to inform; Traditional ambulance"

replace AmbulanceWellEquiped=1 if Ifyesdescribefunctionalityf=="Good status and available fuel sufficiently; Use motor bicycle to inform"

replace AmbulanceWellEquiped=1 if Ifyesdescribefunctionalityf=="Good status and available fuel sufficiently; Use motor bicycle to inform"

replace AmbulanceWellEquiped=1 if Ifyesdescribefunctionalityf=="Good status and available fuel sufficiently; Use motor bicycle to inform"

replace AmbulanceWellEquiped=2 if Ifyesdescribefunctionalityf== "Good status and available fuel sufficiently; Use motor bicycle to inform"

replace AmbulanceWellEquiped = 1 in 2

replace AmbulanceWellEquiped = 1 in 3

replace AmbulanceWellEquiped = 1 in 4

replace AmbulanceWellEquiped=0 if AmbulanceWellEquiped==.

replace AmbulanceWellEquiped = 1 in 6

tab AmbulanceWellEquiped

label variable AmbulanceWellEquiped "Well equiped Ambulance availability"

tab NewbornCorner

tab WeighingscalesNewbornwith50

gen WeighingScalesNewborn=.

replace WeighingScalesNewborn= WeighingscalesNewbornwith50

list WeighingscalesNewbornwith50 WeighingScalesNewborn

tab WeighingScalesNewborn

label variable WeighingScalesNewborn "Weighing scales for Newborn with 50 precision"

label variable WeighingScalesNewborn "Weighing scales for Newborn with 50 precision at one"

replace WeighingScalesNewborn=0 if WeighingScalesNewborn==0

replace WeighingScalesNewborn=1 if WeighingScalesNewborn==1 / WeighingScalesNewborn==4

tab WeighingScalesNewborn

recode WeighingScalesNewborn (0=0) (1/4=1)

tab WeighingScalesNewborn

tab Numberofcompletedeliverysets

gen CompleteDeliverySet=.

replace CompleteDeliverySet= Numberofcompletedeliverysets

tab CompleteDeliverySet

list CompleteDeliverySet Numberofcompletedeliverysets

recode CompleteDeliverySet (0=0) (3/max=1)

tab CompleteDeliverySet

recode CompleteDeliverySet (3=1)

recode CompleteDeliverySet (1/2=0)

drop CompleteDeliverySet

gen CompleteDeliverySet=.

replace CompleteDeliverySet= Numberofcompletedeliverysets

recode CompleteDeliverySet (0/2=0) (3/max=1)

tab CompleteDeliverySet

label variable CompleteDeliverySet "Complete delivery set availability at least three per facility"

tab1 Towelfordryingandwrapping Bulbpigeonsuction Ambubagfacemasksneonatal Mask1 Radiantwarmer

gen TowelDryingWrapping=.

replace TowelDryingWrapping= Towelfordryingandwrapping

recode TowelDryingWrapping (0=0) (2/max=1)

tab Bulbpigeonsuction

gen BulbpigeonsuctionN= Bulbpigeonsuction

tab BulbpigeonsuctionN

recode BulbpigeonsuctionN (0=0) (1/max=1)

gen BulbpigeonsuctionNN= Bulbpigeonsuction

recode BulbpigeonsuctionNN (0/1=0) (2/max=1)

tab BulbpigeonsuctionNN

drop BulbpigeonsuctionN

rename BulbpigeonsuctionNN BulbpigeonsuctionN

label variable TowelDryingWrapping "Towel for drying and wrapping availibility"

label variable BulbpigeonsuctionN "Bulb/pigeon suction availibility at least two per facility"

tab1 TowelDryingWrapping BulbpigeonsuctionN

tab1 Ambubagfacemasksneonatal Mask1 Radiantwarmer

gen Facemasksneonatal0= Ambubagfacemasksneonatal

recode Facemasksneonatal0 (0/1=0) (2/max=1)

gen Facemasksneonatal1= Mask1

recode Facemasksneonatal1 (0/1=0) (2/max=1)

gen RadiantwarmerN= Radiantwarmer

recode RadiantwarmerN (0=0) (1/max=1)

label variable Facemasksneonatal0 "Ambubag with neonatal face mask for neonates size 0)

label variable Facemasksneonatal1 "Ambubag with neonatal face mask for neonates size 1)

label variable RadiantwarmerN "Radiant warmer availability)

tab1 Facemasksneonatal0 Facemasksneonatal1 RadiantwarmerN

label variable RadiantwarmerN "Radiant warmer availability at least one per facility)

label variable Facemasksneonatal1 "Ambubag with neonatal face mask for neonates size 1 at least two per facility)

label variable Facemasksneonatal0 "Ambubag with neonatal face mask for neonates size 0 at least per facility)

tab1 RunningWater Soap

gen RunningWaterN= RunningWater

gen SoapN= Soap

label variable RunningWaterN "Running water availability at delivery room"

label variable SoapN "Soap availability in the delivery ward"

label variable RunningWaterN "Running water availability at delivery ward"

sum DangerSignBPproportion PartographIntiationProportion OxytocinProportion APGARproportion skinToSkinProportion VitKproportion TTCproportion

tab1 DangerSignBPproportion PartographIntiationProportion OxytocinProportion APGARproportion skinToSkinProportion VitKproportion TTCproportion

sum anc4proportion SBAproportion SyphilisProportion PNC48hrsProportion MMR StillBirth NMR24hrs NMR6days PreEclampsiaMgt PROMmgt PPHmgt AsphyxiaMgt SepsisMgt KMC

export excel using "aaaaaa", firstrow(variables)

export excel using "aaaaaa", sheetreplace firstrow(variables)

export excel using "aaaaaa", sheetreplace firstrow(variables)

pca PartographTotal6 OxytocinTotal6 APGARtotal6 skinToSkinTotal6 VitKtotal6 TTCtotal6 anc4proportion SBAproportion SyphilisProportion PNC48hrsProportion MMR StillBirth NMR24hrs NMR6days

screeplot

screeplot

graph matrix PartographTotal6 OxytocinTotal6 APGARtotal6 skinToSkinTotal6 VitKtotal6 TTCtotal6 anc4proportion SBAproportion SyphilisProportion PNC48hrsProportion MMR StillBirth NMR24hrs NMR6days

graph matrix PartographTotal6 OxytocinTotal6 APGARtotal6 skinToSkinTotal6 VitKtotal6 TTCtotal6 anc4proportion SBAproportion SyphilisProportion PNC48hrsProportion MMR StillBirth NMR24hrs NMR6days

tab1 Facilityhasfunctionalincinerat Facilityhasfunctionalplacenta

pca Facilityhasfunctionalincinerat Facilityhasfunctionalplacenta DexamethasonebethamethasoneIVa Methyldopa1stlineavailable Hydralazineavailable Nifedipineavailable AmpicillinIVavailable GentamycinIVavailable MetronidazoleIVavailable TTCeyeointmentavailable VitaminKavailable MgSO4available Calciumgluconateavailable Oxytocinavailable Normalsalineavailable

predict pc1 pc2 pc3 pc4 pc5 pc6

gen yes= pc1+ pc2+ pc3+ pc4+ pc5+ pc6

label variable yes "Scoring value"

tab yes

sum yes

sum yes, detail

gen category=.

replace category=1 if yes>=3.3

replace category=0 if yes<3.3

gen newCategory=.

replace newCategory=1 if DexamethasonebethamethasoneIVa==1& Methyldopa1stlineavailable==1& Hydralazineavailable==1& Nifedipineavailable==1& AmpicillinIVavailable==1& GentamycinIVavailable==1

drop Listhealthfacilitieshealthpo TotalPopulation2008EC TotalPopulation2009EC Healthofficers Nurses Midwives Laboratorypersonnel Pharmacypersonnel

drop BEmONCtrainedandworkingatLD HBBtrainedandworkingatLDor EssentialNewBornCareENBCtra QualityImprovementQualityAssur Hasyourfacilityhadapatients

save "C:\Users\user\Desktop\16. MNH Quality care_Mar 04, 2018.dta", replace

tab1 Doyouusesuggestionbox IfYeshowoftendoyoureviewi Doyouhaveothermeansofgettin Maternaldeathsurveillanceandr Neonataldeathaudits Nearmissaudits

drop FetoscopeDoppler Thermometer Stethoscope Bloodpressureapparatus WeighingscalesAdult

drop WeighingscalesNewbornwith50 Numberofcompletedeliverysets Towelfordryingandwrapping Bulbpigeonsuction Ambubagfacemasksneonatal Mask1 Radiantwarmer Oxygenconcentrator Oxygencylinder RunningWater Soap

drop dangerBP DangerBPpercentage anc1total12 anc4total12 syphilistotal12 skilledBA12 pnc48hrs12 maternalDeath12 stillbirth12 Livebirth12 LBWpreterm12 neonataldeath24hrs12 neonataldeath6days12 preeclampsia12 PROM12 PPH12 AsphyxiaTx12 AsphyxiaCases12 SepsisTx12 SepsisCases12 KMC12 preeclampsiaTx12 PROMTx12 PPHmgt12 ExpectedDelivery12 PartographTotal6 OxytocinTotal6 APGARtotal6 skinToSkinTotal6 VitKtotal6 TTCtotal6

save "C:\Users\user\Desktop\16. MNH Quality care_Mar 04, 2018.dta", replace

save "C:\Users\user\Desktop\16. MNH Quality care_Mar 04, 2018.dta", replace

tab Doesthefacilityhaveelectricp

tab Whatisthesourceofpowersuppl

drop Whatisthesourceofpowersuppl Istherealsobackuppowersuppl howmanyhoursisthepowersuppl Whatisthesourceofwater Isthereabackwaterreservoir IsthereadesignatedspaceforK Isthereanambulancewhichserve Ifyesdescribefunctionalityf

drop MgSO4administrationprotocolava MgSO4administrationprotocoluti BEmONCCEmONCtrainingmanualava BEmONCCEmONCtrainingmanualuti Essentialnewborncareguideline UseEssentialnewborncareguidel Newbornresuscitationflowchart HB Newborncornerguidelineavailabl Newborncornerguidelineutilized AMTSLposteravailable AMTSLposterutilized

drop LQ ANCregisterutilized LabourDeliveryregisterutiliz PNCregisterutilized Referralregisterutilized Stockcardutilized Bincardutilized

tab1 MMR StillBirth NMR24hrs NMR6days

drop MMR NMR24hrs NMR6days

drop Doyouusesuggestionbox IfYeshowoftendoyoureviewi Doyouhaveothermeansofgettin Maternaldeathsurveillanceandr Neonataldeathaudits Perinataldeathaudits Nearmissaudits Functionalcontactnumbersforre FunctionalWellequippedAmbulan Liaisonofficerreferralfocal HastherebeenanyQIinitiative Ifyesprovidebriefdetailorl IsthereaQIteaminthefacilit IfYespleaselistthedesignati Howfrequentlydoestheteammeet Isthereregularsupportivesuper Ifyeshowfrequentitis IfyeshaveyouusedMNHprogram Ifyeswerefeedbacksgiven HowmanymonthlyPHCUreviewmeet WasMNHprogramdiscussed WeretheHEWsparticipated WerethehealthcenterHospital Weretheminutestaken Wasanactionplandevelopedfoll Istheredataqualitycheckingsy Ifyeshowfrequent CompoundcleanlinessLevelofc maternitywardcleanlinessLevel Labourdeliveryroomcleanliness Labourdeliveryroomcleanliness Dotheyusetriplebucklesystem Dotheyusetriplebucklesystem Usethreebucketsforsolidwaste Safetyboxforsharpmaterials

drop Demarcatedsterilizationareafor HIVtestkitsavailable Syphilistestkitsavailable Hemoglobin HaematocritPCV Urinedipstick Urinemicroscopy Microscopyformalaria VDRLRPR BloodGroup

save "C:\Users\user\Desktop\16. MNH Quality care_Mar 04, 2018.dta", replace

tab1 Facilityhasfunctionalincinerat Facilityhasfunctionalplacenta Doesthefacilityhaveelectricp IsthereNewborncornerinthede

drop Normalsalineavailable Ringerslactateavailable

tab1 NewbornCorner

drop IsthereNewborncornerinthede

tab WaterAvailability

tab1 DexamethasonebethamethasoneIVa Methyldopa1stlineavailable Hydralazineavailable Nifedipineavailable AmpicillinIVavailable GentamycinIVavailable MetronidazoleIVavailable TTCeyeointmentavailable VitaminKavailable MgSO4available Calciumgluconateavailable Oxytocinavailable NormalsalineRL

recode NormalsalineRL (1/2=1)

tab NormalsalineRL

pca DangerSignBPproportion PartographIntiationProportion OxytocinProportion APGARproportion skinToSkinProportion VitKproportion TTCproportion

rotate

predict input

predict pca1 pca2

gen inputsScore=.

replace inputsScore= pca1+ pca2

gen InputsScoreN= pca1+ pca2

summarize DangerSignBPproportion PartographIntiationProportion OxytocinProportion APGARproportion skinToSkinProportion VitKproportion TTCproportion

summarize DangerSignBPproportion PartographIntiationProportion OxytocinProportion APGARproportion skinToSkinProportion VitKproportion TTCproportion

corr DangerSignBPproportion PartographIntiationProportion OxytocinProportion APGARproportion skinToSkinProportion VitKproportion TTCproportion

pca DangerSignBPproportion PartographIntiationProportion OxytocinProportion APGARproportion skinToSkinProportion VitKproportion TTCproportion

pca DangerSignBPproportion PartographIntiationProportion OxytocinProportion APGARproportion skinToSkinProportion VitKproportion TTCproportion, mineigen(1)

pca DangerSignBPproportion PartographIntiationProportion OxytocinProportion APGARproportion skinToSkinProportion VitKproportion TTCproportion, comp(1)

pca DangerSignBPproportion PartographIntiationProportion OxytocinProportion APGARproportion skinToSkinProportion VitKproportion TTCproportion, comp(1) blanks(.4)

pca DangerSignBPproportion PartographIntiationProportion OxytocinProportion APGARproportion skinToSkinProportion VitKproportion TTCproportion, comp(1) blanks(.381)

pca DangerSignBPproportion PartographIntiationProportion OxytocinProportion APGARproportion skinToSkinProportion VitKproportion TTCproportion, comp(1) blanks(.3)

factor DangerSignBPproportion PartographIntiationProportion OxytocinProportion APGARproportion skinToSkinProportion VitKproportion TTCproportion

PCA processing_Input

describe OxygenConcentratorOxygenCylinder NewbornCorner AmbulanceWellEquiped WeighingScalesNewborn CompleteDeliverySet TowelDryingWrapping BulbpigeonsuctionN Facemasksneonatal0 Facemasksneonatal1 RadiantwarmerN RunningWaterN SoapN NormalsalineRL WaterAvailability Facilityhasfunctionalincinerat Facilityhasfunctionalplacenta Doesthefacilityhaveelectricp DexamethasonebethamethasoneIVa Methyldopa1stlineavailable Hydralazineavailable Nifedipineavailable AmpicillinIVavailable GentamycinIVavailable MetronidazoleIVavailable TTCeyeointmentavailable VitaminKavailable MgSO4available Calciumgluconateavailable Oxytocinavailable

summarize OxygenConcentratorOxygenCylinder NewbornCorner AmbulanceWellEquiped WeighingScalesNewborn CompleteDeliverySet TowelDryingWrapping BulbpigeonsuctionN Facemasksneonatal0 Facemasksneonatal1 RadiantwarmerN RunningWaterN SoapN NormalsalineRL WaterAvailability Facilityhasfunctionalincinerat Facilityhasfunctionalplacenta Doesthefacilityhaveelectricp DexamethasonebethamethasoneIVa Methyldopa1stlineavailable Hydralazineavailable Nifedipineavailable AmpicillinIVavailable GentamycinIVavailable MetronidazoleIVavailable TTCeyeointmentavailable VitaminKavailable MgSO4available Calciumgluconateavailable Oxytocinavailable

corr OxygenConcentratorOxygenCylinder NewbornCorner AmbulanceWellEquiped WeighingScalesNewborn CompleteDeliverySet TowelDryingWrapping BulbpigeonsuctionN Facemasksneonatal0 Facemasksneonatal1 RadiantwarmerN RunningWaterN SoapN NormalsalineRL WaterAvailability Facilityhasfunctionalincinerat Facilityhasfunctionalplacenta Doesthefacilityhaveelectricp DexamethasonebethamethasoneIVa Methyldopa1stlineavailable Hydralazineavailable Nifedipineavailable AmpicillinIVavailable GentamycinIVavailable MetronidazoleIVavailable TTCeyeointmentavailable VitaminKavailable MgSO4available Calciumgluconateavailable Oxytocinavailable

pca Facilityhasfunctionalincinerat Facilityhasfunctionalplacenta Doesthefacilityhaveelectricp WaterAvailability DexamethasonebethamethasoneIVa Methyldopa1stlineavailable Hydralazineavailable Nifedipineavailable AmpicillinIVavailable GentamycinIVavailable MetronidazoleIVavailable TTCeyeointmentavailable VitaminKavailable MgSO4available Calciumgluconateavailable Oxytocinavailable NormalsalineRL OxygenConcentratorOxygenCylinder NewbornCorner AmbulanceWellEquiped WeighingScalesNewborn CompleteDeliverySet TowelDryingWrapping BulbpigeonsuctionN Facemasksneonatal0 Facemasksneonatal1 RadiantwarmerN RunningWaterN SoapN

screeplot

screeplot, yline(1)

pca Facilityhasfunctionalincinerat Facilityhasfunctionalplacenta Doesthefacilityhaveelectricp WaterAvailability DexamethasonebethamethasoneIVa Methyldopa1stlineavailable Hydralazineavailable Nifedipineavailable AmpicillinIVavailable GentamycinIVavailable MetronidazoleIVavailable TTCeyeointmentavailable VitaminKavailable MgSO4available Calciumgluconateavailable Oxytocinavailable NormalsalineRL OxygenConcentratorOxygenCylinder NewbornCorner AmbulanceWellEquiped WeighingScalesNewborn CompleteDeliverySet TowelDryingWrapping BulbpigeonsuctionN Facemasksneonatal0 Facemasksneonatal1 RadiantwarmerN RunningWaterN SoapN, mineigen(1)

pca Facilityhasfunctionalincinerat Facilityhasfunctionalplacenta Doesthefacilityhaveelectricp WaterAvailability DexamethasonebethamethasoneIVa Methyldopa1stlineavailable Hydralazineavailable Nifedipineavailable AmpicillinIVavailable GentamycinIVavailable MetronidazoleIVavailable TTCeyeointmentavailable VitaminKavailable MgSO4available Calciumgluconateavailable Oxytocinavailable NormalsalineRL OxygenConcentratorOxygenCylinder NewbornCorner AmbulanceWellEquiped WeighingScalesNewborn CompleteDeliverySet TowelDryingWrapping BulbpigeonsuctionN Facemasksneonatal0 Facemasksneonatal1 RadiantwarmerN RunningWaterN SoapN, comp(10)

pca Facilityhasfunctionalincinerat Facilityhasfunctionalplacenta Doesthefacilityhaveelectricp WaterAvailability DexamethasonebethamethasoneIVa Methyldopa1stlineavailable Hydralazineavailable Nifedipineavailable AmpicillinIVavailable GentamycinIVavailable MetronidazoleIVavailable TTCeyeointmentavailable VitaminKavailable MgSO4available Calciumgluconateavailable Oxytocinavailable NormalsalineRL OxygenConcentratorOxygenCylinder NewbornCorner AmbulanceWellEquiped WeighingScalesNewborn CompleteDeliverySet TowelDryingWrapping BulbpigeonsuctionN Facemasksneonatal0 Facemasksneonatal1 RadiantwarmerN RunningWaterN SoapN, comp(10) blank(.3)

pca Facilityhasfunctionalincinerat Facilityhasfunctionalplacenta Doesthefacilityhaveelectricp WaterAvailability DexamethasonebethamethasoneIVa Methyldopa1stlineavailable Hydralazineavailable Nifedipineavailable AmpicillinIVavailable GentamycinIVavailable MetronidazoleIVavailable TTCeyeointmentavailable VitaminKavailable MgSO4available Calciumgluconateavailable Oxytocinavailable NormalsalineRL OxygenConcentratorOxygenCylinder NewbornCorner AmbulanceWellEquiped WeighingScalesNewborn CompleteDeliverySet TowelDryingWrapping BulbpigeonsuctionN Facemasksneonatal0 Facemasksneonatal1 RadiantwarmerN RunningWaterN SoapN, comp(10) blank(.25)

pca Facilityhasfunctionalincinerat Facilityhasfunctionalplacenta Doesthefacilityhaveelectricp WaterAvailability DexamethasonebethamethasoneIVa Methyldopa1stlineavailable Hydralazineavailable Nifedipineavailable AmpicillinIVavailable GentamycinIVavailable MetronidazoleIVavailable TTCeyeointmentavailable VitaminKavailable MgSO4available Calciumgluconateavailable Oxytocinavailable NormalsalineRL OxygenConcentratorOxygenCylinder NewbornCorner AmbulanceWellEquiped WeighingScalesNewborn CompleteDeliverySet TowelDryingWrapping BulbpigeonsuctionN Facemasksneonatal0 Facemasksneonatal1 RadiantwarmerN RunningWaterN SoapN, comp(10) blank(.27)

rotate, varimax

rotate, varimax blanks(.27)

rotate, promax

rotate, promax blanks(.27)

rotate, clear

loadingplot

scoreplot

estat loadings

predict pc1 pc2 pc3 pc4 pc5 pc6 pc7 pc8 pc9 pc10, score

estat kmo

gen InputScore= pc1+ pc2+ pc3+ pc4+ pc5+ pc6+ pc7+ pc8+ pc9+ pc10

describe InputScore

summarize InputScore

alpha Facilityhasfunctionalincinerat Facilityhasfunctionalplacenta Doesthefacilityhaveelectricp WaterAvailability DexamethasonebethamethasoneIVa Methyldopa1stlineavailable Hydralazineavailable Nifedipineavailable AmpicillinIVavailable GentamycinIVavailable MetronidazoleIVavailable TTCeyeointmentavailable VitaminKavailable MgSO4available Calciumgluconateavailable Oxytocinavailable NormalsalineRL OxygenConcentratorOxygenCylinder NewbornCorner AmbulanceWellEquiped WeighingScalesNewborn CompleteDeliverySet TowelDryingWrapping BulbpigeonsuctionN Facemasksneonatal0 Facemasksneonatal1 RadiantwarmerN RunningWaterN SoapN

PCA processing_Process

describe DangerSignBPproportion PartographIntiationProportion OxytocinProportion APGARproportion skinToSkinProportion VitKproportion TTCproportion

summarize DangerSignBPproportion PartographIntiationProportion OxytocinProportion APGARproportion skinToSkinProportion VitKproportion TTCproportion

corr DangerSignBPproportion PartographIntiationProportion OxytocinProportion APGARproportion skinToSkinProportion VitKproportion TTCproportion

alpha DangerSignBPproportion PartographIntiationProportion OxytocinProportion APGARproportion skinToSkinProportion VitKproportion TTCproportion

pca DangerSignBPproportion PartographIntiationProportion OxytocinProportion APGARproportion skinToSkinProportion VitKproportion TTCproportion

screeplot

screeplot, yline(1)

pca DangerSignBPproportion PartographIntiationProportion OxytocinProportion APGARproportion skinToSkinProportion VitKproportion TTCproportion, mineigen(.8)

pca DangerSignBPproportion PartographIntiationProportion OxytocinProportion APGARproportion skinToSkinProportion VitKproportion TTCproportion, comp(2)

pca DangerSignBPproportion PartographIntiationProportion OxytocinProportion APGARproportion skinToSkinProportion VitKproportion TTCproportion, comp(1) blanks(.3)

pca DangerSignBPproportion PartographIntiationProportion OxytocinProportion APGARproportion skinToSkinProportion VitKproportion TTCproportion, comp(2) blanks(.3)

rotate, varimax

rotate, varimax blanks(.3)

rotate, promax

rotate, promax blanks(.3)

rotate, clear

loadingplot

scoreplot

estat loadings

predict pca1 pca2, score

estat kmo

xtile per = ProcessScore, nquantiles(4) altdef

gen ProcessScoreCat=.

replace ProcessScoreCat=1 if ProcessScore>=1.8

replace ProcessScoreCat=0 if ProcessScore<1.8

label define ProcessScoreCat 1 "Good process Quality" 0 "Not Good Process Quality"

Simple scoring

gen InfrustScore= (Facilityhasfunctionalincinerat+ Facilityhasfunctionalplacenta+ Doesthefacilityhaveelectricp+ WaterAvailability)/4

label variable InfrustScore "Infrustructure input score"

label variable InputScore "Input score using Principal components"

label variable ProcessScore "Process score using Principal components"

label variable ProcessScoreCat "Process score category using quartiles"

gen EquipSupplyScore= ( OxygenConcentratorOxygenCylinder+ NewbornCorner+ AmbulanceWellEquiped+ WeighingScalesNewborn+ CompleteDeliverySet+ TowelDryingWrapping+ BulbpigeonsuctionN+ Facemasksneonatal0+ Facemasksneonatal1+ RadiantwarmerN+ RunningWaterN)/11

label variable EquipSupplyScore "Equipment and supply input score"

sum InfrustScore

sum EquipSupplyScore

gen DrugsScore= (DexamethasonebethamethasoneIVa+ Methyldopa1stlineavailable+ Hydralazineavailable+ Nifedipineavailable+ AmpicillinIVavailable+ GentamycinIVavailable+ MetronidazoleIVavailable+ TTCeyeointmentavailable+ VitaminKavailable+ MgSO4available+ Calciumgluconateavailable+ Oxytocinavailable+ NormalsalineRL)/13

des DrugsScore

sum DrugsScore

gen OverAllInputs= (DexamethasonebethamethasoneIVa+ Methyldopa1stlineavailable+ Hydralazineavailable+ Nifedipineavailable+ AmpicillinIVavailable+ GentamycinIVavailable+ MetronidazoleIVavailable+ TTCeyeointmentavailable+ VitaminKavailable+ MgSO4available+ Calciumgluconateavailable+ Oxytocinavailable+ NormalsalineRL+OxygenConcentratorOxygenCylinder+ NewbornCorner+ AmbulanceWellEquiped+ WeighingScalesNewborn+ CompleteDeliverySet+ TowelDryingWrapping+ BulbpigeonsuctionN+ Facemasksneonatal0+ Facemasksneonatal1+ RadiantwarmerN+ RunningWaterN+ Facilityhasfunctionalincinerat+ Facilityhasfunctionalplacenta+ Doesthefacilityhaveelectricp+ WaterAvailability)/29

des OverAllInputs

sum OverAllInputs

label variable DrugsScore "Essential drugs score"

label variable OverAllInputs "Overall Inputs score"

gen OverAllInputsCat=.

replace OverAllInputsCat=1 if OverAllInputs>=.75

replace OverAllInputsCat=1 if OverAllInputs>=.6

replace OverAllInputsCat=0 if OverAllInputs<.6

tab OverAllInputsCat

label variable OverAllInputsCat "Over All Input Score Category"

tab OverAllInputsCat

replace PROMmgt=0 if PROMmgt==.

replace PreEclampsiaMgt=0 if PreEclampsiaMgt==.

replace PPHmgt=0 if PPHmgt==.

replace AsphyxiaMgt=0 if AsphyxiaMgt==.

replace SepsisMgt=0 if SepsisMgt==.

replace KMC=0 if KMC==.

gen ClinicalService= DangerSignBPproportion+ PartographIntiationProportion+ OxytocinProportion+ APGARproportion+ skinToSkinProportion+ VitKproportion+ TTCproportion

gen ComplicatinMgt= PreEclampsiaMgt+ PROMmgt+ PPHmgt+ AsphyxiaMgt+ SepsisMgt+ KMC

gen OverAllProcesScore= (PreEclampsiaMgt+PROMmgt+ PPHmgt+ AsphyxiaMgt+ SepsisMgt+ KMC+ DangerSignBPproportion+ PartographIntiationProportion+ OxytocinProportion+ APGARproportion+ skinToSkinProportion+ VitKproportion+ TTCproportion)/13

tab1 ClinicalService ComplicatinMgt OverAllProcesScore

sum ClinicalService ComplicatinMgt OverAllProcesScore

gen ClinicalService7= ClinicalService/7

gen ComplicationMgt6= ComplicatinMgt/6

graph hbar (mean) PreEclampsia (mean) PROMmgt (mean) PPHmgt (mean) AsphyxiaMgt (mean) SepsisMgt (mean) KMC, showyvars bargap(10) blabel(total, format(%4.2g)) ytitle(Proportion) ylabel(, labels valuelabel) title(Proportion of complication management in Health facilities) legend(off) clegend(on)

bysort TypeofFacility: sum OverAllInputsCat

bysort TypeofFacility: sum OverAllInputs

gen OutPutScore= (anc4proportion+ SBAproportion+ SyphilisProportion+ PNC48hrsProportion)/4

drop pc1 pc2 pc3 pc4 pc5 pc6 pc7 pc8 pc9 pc10 InputScore pca1 pca2 ProcessScore per ProcessScoreCat

label variable compnscore "Complication managment Score"

label variable OutPutScore "Output Score"

sum OverAllInputs OverAllProcesScore OutPutScore

bysort TypeofFacility: sum OverAllInputs OverAllProcesScore OutPutScore

gen OverAllInputCat=.

replace OverAllInputCat=1 if OverAllInputs>=.75

replace OverAllInputCat=0 if OverAllInputs<.75

label variable OverAllInputCat "Over all Input Score"

gen OverAllProcessCat=.

replace OverAllProcessCat=1 if OverAllProcesScore>=.75

replace OverAllProcessCat=0 if OverAllProcesScore<.75

gen OverAllOutputCat=.

replace OverAllOutputCat =1 if OutPutScore >=.75

replace OverAllOutputCat =0 if OutPutScore <.75

label variable OverAllProcessCat "Over all Process Score Category"

label variable OverAllInputCat "Over all Input Score Category"

label variable OverAllOutputCat "Over all Output Score Category"

bysort TypeofFacility: sum OverAllInputCat OverAllProcessCat OverAllOutputCat

bysort TypeofFacility: tab1 OverAllInputCat OverAllProcessCat OverAllOutputCat

replace OverAllOutputCat = . in 25

replace OverAllOutputCat = . in 31

replace OverAllOutputCat = . in 29

bysort TypeofFacility: tab1 OverAllInputCat OverAllProcessCat OverAllOutputCat

gen AllOutputProcessInput= OverAllInputCat+ OverAllProcessCat+ OverAllOutputCat

label variable AllOutputProcessInput "All components result"

use "C:\Users\dell\Desktop\20. MNH Quality care_Mar 20, 2018.dta"

gen InfrastructureAll

gen InfrastructureAll = Facilityhasfunctionalincinerat + Facilityhasfunctionalplacenta + Doesthefacilityhaveelectricp + RunningWaterN

label variable InfrastructureAll "Proportion of health facilities with all infrastructure inputs available"

summarize InfrastructureAll

replace InfrastructureAll = 0 if InfrastructureAll <4

replace InfrastructureAll = 1 if InfrastructureAll >=4

save "C:\Users\dell\Desktop\20. MNH Quality care_Mar 20, 2018.dta", replace

gen DrugAll = DexamethasonebethamethasoneIVa+ Methyldopa1stlineavailable+ Hydralazineavailable+ Nifedipineavailable+ AmpicillinIVavailable+ GentamycinIVavailable+ MetronidazoleIVavailable+ TTCeyeointmentavailable+ VitaminKavailable+ MgSO4available+ Calciumgluconateavailable+ Oxytocinavailable+ NormalsalineRL

label variable DrugAll "Proportion of health facilities with all drugs inputs available"

summarize DrugAll

replace DrugAll = 0 if DrugAll <13

replace DrugAll = 0 if DrugAll >=13

gen EquipmentsSupplyAll = OxygenConcentratorOxygenCylinder+ NewbornCorner+ AmbulanceWellEquiped+ WeighingScalesNewborn+ CompleteDeliverySet+ TowelDryingWrapping+ BulbpigeonsuctionN+ Facemasksneonatal0+ Facemasksneonatal1+ RadiantwarmerN+ RunningWaterN

summarize EquipmentsSupplyAll

replace EquipmentsSupplyAll l = 0 if EquipmentsSupplyAll <11

replace EquipmentsSupplyAll = 0 if EquipmentsSupplyAll <11

replace EquipmentsSupplyAll = 0 if EquipmentsSupplyAll >=11

label variable EquipmentsSupplyAll "Proportion of health facilities with all Equipment/supply inputs available"
